# Alcoholic Liver Disease: Alcohol Metabolism, Cascade of Molecular Mechanisms, Cellular Targets, and Clinical Aspects

**DOI:** 10.3390/biomedicines6040106

**Published:** 2018-11-12

**Authors:** Rolf Teschke

**Affiliations:** Department of Internal Medicine II, Division of Gastroenterology and Hepatology, Klinikum Hanau, Leimenstrasse 20, D-63450 Hanau, Academic Teaching Hospital of the Medical Faculty, Goethe University Frankfurt/Main, Frankfurt/Main, Germany; rolf.teschke@gmx.de; Tel.: +49-618-1/21-859; Fax: +49-618-1/29-64211

**Keywords:** MEOS, microsomal ethanol-oxidizing system, alcohol dehydrogenase, ethanol, acetaldehyde, alcohol metabolism, alcoholic liver disease, alcoholic fatty liver, alcoholic steatohepatitis, alcoholic hepatitis, alcoholic cirrhosis, hepatocellular carcinoma, circadian rhythms, CYP 2E1, endotoxins, intestinal microbiome, SIRT, ROS

## Abstract

Alcoholic liver disease is the result of cascade events, which clinically first lead to alcoholic fatty liver, and then mostly via alcoholic steatohepatitis or alcoholic hepatitis potentially to cirrhosis and hepatocellular carcinoma. Pathogenetic events are linked to the metabolism of ethanol and acetaldehyde as its first oxidation product generated via hepatic alcohol dehydrogenase (ADH) and the microsomal ethanol-oxidizing system (MEOS), which depends on cytochrome P450 2E1 (CYP 2E1), and is inducible by chronic alcohol use. MEOS induction accelerates the metabolism of ethanol to acetaldehyde that facilitates organ injury including the liver, and it produces via CYP 2E1 many reactive oxygen species (ROS) such as ethoxy radical, hydroxyethyl radical, acetyl radical, singlet radical, superoxide radical, hydrogen peroxide, hydroxyl radical, alkoxyl radical, and peroxyl radical. These attack hepatocytes, Kupffer cells, stellate cells, and liver sinusoidal endothelial cells, and their signaling mediators such as interleukins, interferons, and growth factors, help to initiate liver injury including fibrosis and cirrhosis in susceptible individuals with specific risk factors. Through CYP 2E1-dependent ROS, more evidence is emerging that alcohol generates lipid peroxides and modifies the intestinal microbiome, thereby stimulating actions of endotoxins produced by intestinal bacteria; lipid peroxides and endotoxins are potential causes that are involved in alcoholic liver injury. Alcohol modifies SIRT1 (Sirtuin-1; derived from Silent mating type Information Regulation) and SIRT2, and most importantly, the innate and adapted immune systems, which may explain the individual differences of injury susceptibility. Metabolic pathways are also influenced by circadian rhythms, specific conditions known from living organisms including plants. Open for discussion is a 5-hit working hypothesis, attempting to define key elements involved in injury progression. In essence, although abundant biochemical mechanisms are proposed for the initiation and perpetuation of liver injury, patients with an alcohol problem benefit from permanent alcohol abstinence alone.

## 1. Introduction

Alcohol is chemically ethyl alcohol, or in condensed form described as ethanol, whereby these terms are often used interchangeably in the clinical context. Ethanol, a short-chain hydrocarbon C_2_H_5_OH, has a more non-polar chemical structure, and it is thereby water soluble, but less soluble in lipids with their polar molecules. This ambiguity of polarity facilitates a rapid diffusion of ethanol through biological membranes, allowing at the same time changes in the membrane properties, whereas the mechanism of action remains controversial [1,2]. Ethanol may target the bilayer structures of outer cell membranes or the monolayer membranes of organelles such as mitochondria or the endoplasmic reticulum, considering that both membrane types contain lipids preferentially as phospholipids. There is also evidence for direct alcohol interactions with membrane proteins [3]. This may have an additional impact on functional and injurious changes in organs that are affected by chronic alcohol consumption. Therefore, alcohol related organ injuries and functional alterations could unquestionably be classified as molecular, membrane-targeted diseases, a definition that is well applicable also to alcoholic liver disease (ALD).

Among various liver diseases such as drug-induced liver injury (DILI), herb-induced liver injury (HILI), nonalcoholic fatty liver disease (NAFLD), nonalcoholic steatohepatitis (NASH), or those caused by hepatitis B virus (HBV) and hepatitis C virus (HCV) infections, especially ALD continues to attract much interest from scientists and clinicians worldwide [4,5,6,7,8,9,10,11,12,13,14,15]. Their stimulating and partially controversial discussions focused on the pathogenetic aspects [4,5], clinical features [6], and therapeutic approaches [7,8,9] including liver transplantation [10,11,12,13]. Consensus exists that ALD contributes significantly to the global burden of mortality [14,15]. In 2010, alcoholic cirrhosis (AC) was globally responsible for around 493,300 deaths, corresponding to around 0.9% of all global deaths [15].

This review article provides an update on a few relevant issues of alcohol and acetaldehyde metabolism, molecular and cellular mechanisms leading to liver injury, clinical features, and diagnostic approaches, as well as therapeutic modalities, including the option for liver transplantation. It is well recognized that there exist myriads of alcoholic studies with several hundreds of molecular mechanisms, and many mediators are provided by expert biochemists rather than a satisfactory unifying mechanistic approach that is considered valid for a sound discussion of a yet partially controversial clinical topic.

## 2. Data Search and Source

The PubMed database was used to identify publications for the following terms: Alcoholic liver disease, alcoholic fatty liver disease, alcoholic steatohepatitis, alcoholic hepatitis, alcoholic cirrhosis, alcohol metabolism, alcohol dehydrogenase, microsomal ethanol-oxidizing system, cytochrome P450 2E1 (CYP 2E1), catalase, and mitochondrial acetaldehyde dehydrogenase. Publications of the first 50 hits from each searched segment were analyzed. The search was completed on 24 September 2018. Prior to the final analysis, the publications were assessed regarding clinical quality and data completeness. The final selection of publications was restricted to those in English language to ensure transparent accessibility. 

## 3. Alcohol Absorption 

Alcoholic beverages are commonly nature-based products, initially involving photosynthesis in the plants [16,17]. Glucose is produced, a chemical ingredient of many fruits such as grapes, used as source to produced wine, a process known in many countries since ancient times. During wine production and fermentation, glucose is converted to alcohol. Following ingestion, maximum blood alcohol levels are higher if alcohol is consumed with an empty stomach [18], if highly concentrated alcoholic beverages such as spirits were used within a short time without a meal [19], or if patients had a past medical history (PMH) of gastrectomy surgery causing rapid intestinal alcohol uptake [20]. 

## 4. Gastric Alcohol Dehydrogenase and First-Pass Metabolism of Alcohol

Although the liver is the preferred organ of alcohol degradation, it is metabolized in small amounts by the gastric mucosa, which contains alcohol dehydrogenase (ADH), a process called gastric first-pass metabolism (FPM) [20,21,22].

## 5. Hepatic Alcohol Metabolism

In the liver, three different enzymes are known, which in vitro can metabolize ethanol to acetaldehyde [22,23,24,25]. These are ADH, the microsomal ethanol-oxidizing system (MEOS), and catalase:

ADH: C_2_H_5_OH + NAD^+^ → CH_3_CHO + NADH + H^+^ Ethanol     Acetaldehyde

MEOS: C_2_H_5_OH + NADPH + H^+^ + O_2_ → CH_3_CHO + NADP^+^ + 2H_2_O Ethanol          Acetaldehyde

Catalase: C_2_H_5_OH + H_2_O_2_ → CH_3_CHO + 2H_2_O   Ethanol     Acetaldehyde

**Figure 1:**
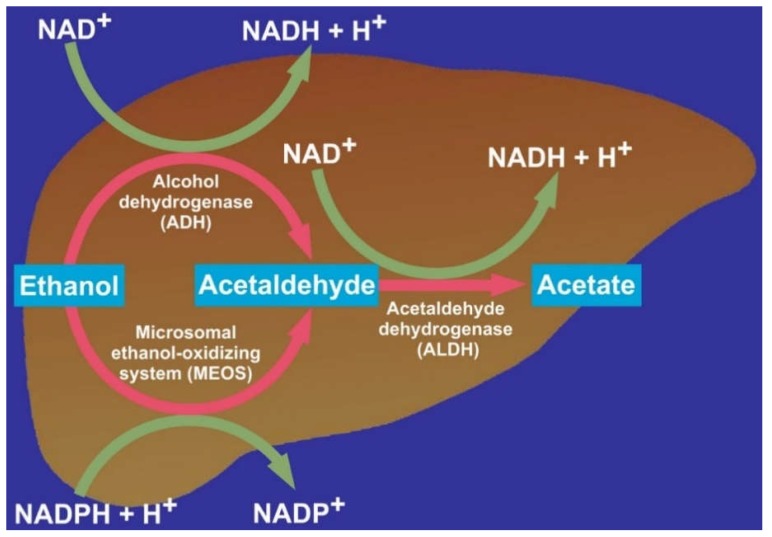
Significant pathways of hepatic alcohol and acetaldehyde metabolism. For alcohol metabolism, presented are cytosolic alcohol dehydrogenase (ADH) and the microsomal ethanol-oxidizing system (MEOS); both pathways metabolize ethanol to acetaldehyde. Reproduced from a previous report [25], with permission of the Publisher Taylor & Francis (Didcot, UK).

Within the liver cell, ADH is found in the cytoplasm (cytosol) between the various subcellular structures, MEOS in the endoplasmic reticulum, and catalase in the peroxisomes, also called microbodies. Of relevance for hepatic alcohol metabolism in vivo are ADH and MEOS but not catalase (Figure 1) [22,23,24,25].

## 6. Hepatic Alcohol Dehydrogenase

Hepatic ADH (EC 1.1.1.1) is a well-studied enzyme of the hepatocyte cytosol. Several aspects of hepatic ADH have been discussed in various reports [22,23,24,25]. The mammalian ADHs represent a group of enzymes that catalyze the oxidation and reduction of a wide variety of alcohols and aldehydes, with individual differences in ADH isozymes. 

Human liver ADH consists of five classes, ADH1 through ADH5, characterized by individual subunits. For class I, the subunits α, β, and γ are described [22,24]. Polymorphism is a characteristic feature of ADH2 and ADH3 that encode the β and γ subunits. ADH2 isoenzymes migrate more anodically than ADH1 isoenzymes, and have a relatively high *K*_m_ for ethanol (34 mM). ADH can easily be differentiated form the other enzymes metabolizing alcohol, MEOS, and catalase (Table 1). 

In animal studies, ADH was characterized and differentiated from MEOS and catalase [22,23,25,26,27,28,29]. ADH is active at a pH optimum of 11, and it has a *K*_m_ for ethanol of 0.5–2.0 mM, corresponding to 0.025–0.1‰ ethanol. The high pH optimum of ADH in animals is substantially above the physiological pH of 7.4, conditions that may limit the role of hepatic ADH in experimental studies of alcohol metabolism. However, its low *K*_m_ values for ethanol are theoretically in favor of a major role of ADH in experimental hepatic alcohol metabolism, but only at low and not at higher alcohol concentrations. Since hepatic ADH activity is not inducible after chronic alcohol use, this enzyme cannot account for the increased alcohol metabolism observed after prolonged alcohol intake. Estimates of the role of hepatic ADH in alcohol metabolism of non-alcoholic humans will remain a matter of debate, due to the variability of genetic ADH isoenzymes as confounding variables.

## 7. Hepatic Microsomal Ethanol-Oxidizing System

The discovery of MEOS by the pioneering scientists Charles S. Lieber and Leonore M. DeCarli was published in 1968 [26], followed by their detailed characterization of MEOS, reported in 1970 [27,28], and its tentative role for ethanol metabolism in vivo, published in 1972 [29]. Subsequent publications focused on this new pathway of alcohol metabolism and related clinical aspects [30,31,32,33,34,35,36,37,38,39,40,41,42,43,44,45,46,47,48,49,50,51,52,53,54,55,56,57,58,59,60,61,62,63,64,65,66,67,68,69,70,71,72,73,74,75,76,77,78,79,80,81,82,83,84,85,86,87,88,89,90,91,92,93,94,95,96,97,98,99,100,101,102,103,104,105,106,107,108,109,110,111,112,113,114,115,116,117,118,119,120,121,122,123,124,125,126,127,128,129,130,131,132,133,134,135,136,137,138,139,140,141,142,143,144,145,146,147,148,149,150,151,152,153,154,155,156,157,158,159,160,161,162,163,164,165,166,167,168,169,170,171,172,173,174,175,176,177,178,179,180,181,182,183,184,185,186,187,188,189,190,191,192,193,194,195,196,197,198,199,200,201,202,203,204,205,206,207,208,209,210,211,212,213,214,215,216,217,218,219,220,221,222,223,224,225,226,227,228,229,230,231,232,233,234,235,236,237,238,239,240,241,242,243]. In addition, various review articles discussed new developments of MEOS, including its constituents such as cytochrome P450 (CYP), or more specifically, its isoenzyme, CYP 2E1 [23,25,41,45,52,53,54,55,75,102,234,244,245,246,247,248,249,250,251,252]. The discovery of MEOS [26,27,248,250] goes back to an ultrastructural study, which revealed a striking centrilobular and midzonal proliferation of the smooth endoplasmic reticulum in the livers of rats after consumption of a liquid diet containing ethanol [253]. These ultrastructural changes were associated with a typical alcoholic fatty liver [253], and could be reproduced in volunteers given alcohol under controlled metabolic ward conditions [254]. Such careful observations in experimental and human studies finally facilitated the discovery of MEOS [26,27,28,29,250].

The nature of the newly described MEOS was heavily debated. In particular, there were initial claims of others that this system may be accounted for by enzymes contaminating the liver microsomal fraction during the preparative procedures. The specific critical focus was on ADH, catalase, or both together as possible causative contaminants. However, yet the initially described characteristics of MEOS were clearly different from those of ADH and catalase [26,27,28], with various differentiating features as listed in detail (Table 1). Such possible contaminants had early been considered in the initial publications describing MEOS as a unique enzyme system, but their role in MEOS was clarified using specific inhibitors of ADH and catalase, which showed that MEOS activities remained virtually unchanged under these inhibitory conditions [26,27].

**Figure 2:**
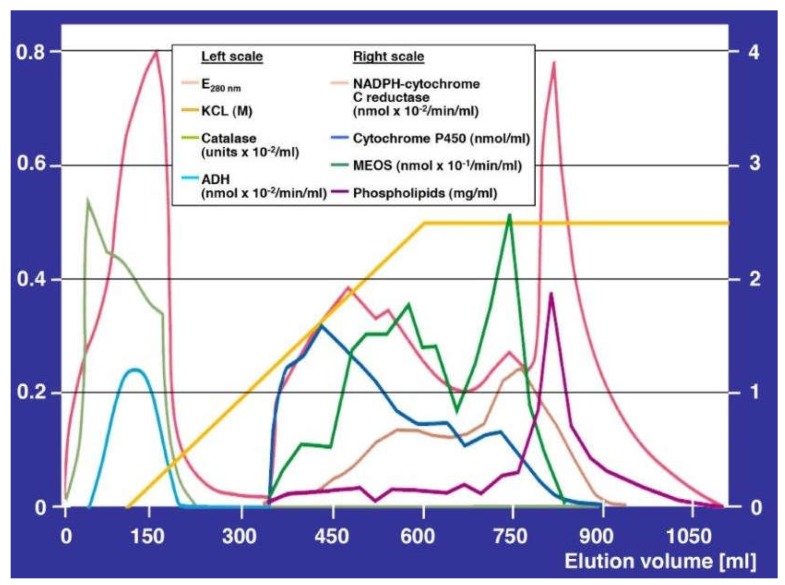
Purification of the microsomal ethanol-oxidizing system (MEOS) and its separation from catalase and alcohol dehydrogenase (ADH) activities. Separation was achieved by DEAE (Diethyl-Amino-Ethyl) cellulose ion exchange column chromatography after solubilization of liver microsomes obtained from rats fed an ethanol containing liquid diet for three weeks. In the void volume eluted up to around 220 mL, the highest peak represents the protein curve assessed as E_280 nm_, and the peak below that is the catalase peak, whereas ADH presents as the lowest peak. Starting with an elution volume of around 330 mL, microsomal components begin to appear. The first peak represents cytochrome P450, the second peak represents E_280 nm_, followed by a third peak with two shoulders and by a fourth peak representing MEOS. At around 770 mL, the reductase peak emerges, followed by the phospholipid peak at around 790 mL elution volume. Overall, this experimental approach was challenging, putting active MEOS on the top of the column and expecting active MEOS in the effluents. There was a high risk of inactivation of MEOS, not only during the solubilization procedure using ultrasonication and deoxycholate that disintegrated MEOS out of the intact microsomal membranes, but also during the chromatography procedure itself that could lead to the inactive cytochrome P420 from the active P450. The original figure was published in a previous report [30] and is reproduced with permission of the Publisher Elsevier (Amsterdam, The Netherlands).

A step forward was the physical separation of MEOS in rat liver from both ADH and catalase activities by DEAE (Diethyl-Amino-Ethyl) cellulose ion exchange column chromatography [30]. MEOS likely consists of cytochrome P450 (CYP), NADPH-cytochrome P450 reductase, and phospholipids (Figure 2).

**Figure 3:**
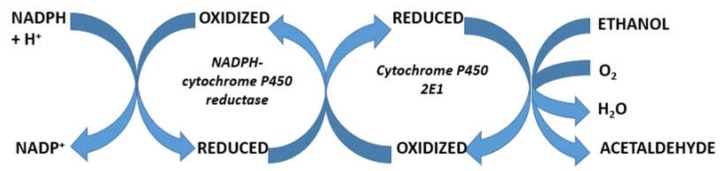
Constituents of MEOS. A key role is attributed to the hepatic microsomal cytochrome P450 2E1, but NADPH-cytochrome P450 reductase plays also an obligatory role; the metabolic reaction of MEOS requires phospholipids, the site of their reaction is unknown. Reproduced from a previous report [25], with permission of the Publisher Taylor & Francis (Didcot, UK).

Based on the observation that MEOS activity was found only in column fractions rich in CYP, this suggested that MEOS is an enzyme system that depends strongly on cytochrome P450 (Figure 3) [30]. 

Of note, the previously described and partially purified ethanol-inducible cyanide binding cytochrome [51,255,256] is presumably identical to the later described ethanol-inducible CYP 2E1 as the most important isoenzyme (Table 2) [192,245,246,247,248,249,250]. 

**Figure 4:**
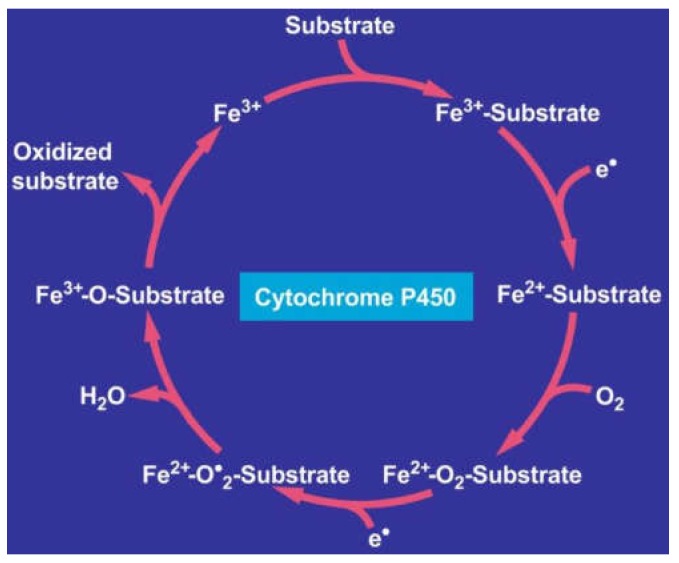
Hepatic microsomal cytochrome P450 and its interaction with substrates. Cytochrome P450 catalyzes the oxidation of substrates such as drugs and ethanol, which bind to the ferric (3^+^) iron of the cytochrome P450 as the initial metabolic step leading finally to the oxidized substrate. The original figure was published in a recent article [257].

Reconstitution experiments supported the concept that MEOS consists of cytochrome P450, with preference of its isoenzyme CYP 2E1, the reductase, and phospholipids [49,52,55,57]. Other investigators reproduced our initial separation studies [78] published before [30] and thereby confirmed that MEOS is indeed an enzyme system that is independent of ADH and catalase [57,78]. Finally, CYP 2E1 was purified and characterized in human liver as a hemeprotein with a molecular weight of 54 daltons, which oxidizes ethanol at a turnover rate of 12.2 nmole min^−1^ in a reconstituted system [110]. The capacity of the ethanol-inducible CYP 2E1 to generate reactive oxygen species (ROS) such as superoxide and hydroxyl radicals has been published in several reports [50,56,92,152]. In analogy to many drugs and chemicals, ethanol functions as a substrate for microsomal CYP through an oxidation process, which may partially be incomplete and thereby produce reactive oxygen forms (Figure 4) [257]. 

The reactions within the CYP cycle involve the uptake of two electrons and molecular oxygen, yielding the oxidized substrate plus water (Figure 4). The iron in the cytochrome P450 molecule returns to its ferric (3^+^) state, after having been in a ferrous state (2^+^), and is again available for binding to the next substrate molecule. AS part of usual life in any healthy organism, radicals are continuously generated through incomplete intracellular oxygen splitting, and are commonly scavenged by antioxidants.

Whereas MEOS activity is induced by chronic alcohol consumption as established by several studies [26,27,28,29,30], even a single dose of ethanol can increase MEOS activity [76]. The induction of MEOS activity was found to be modulated by the composition of the alcohol diets containing various amounts of fat or carbohydrates [40,68,137]. For instance, the induction of MEOS activity was facilitated by a high-fat diet [40] or a low-carbohydrate diet [68]. However, under an alcohol diet with a high amount of carbohydrates the usual alcohol-dependent induction of MEOS activity was offset [68]. Similar to MEOS, hepatic microsomal CYP 2E1 is regulated by dietary lipids and carbohydrates [137]. There is now also sufficient evidence that the induction of the ethanol-specific CYP 2E1 is the result of increased enzyme synthesis [153], found in the course of prolonged ethanol use, and explained via transcription of the *CYP 2E1* gene at high alcohol values in the blood [156].

Based on animal studies, it was early recognized that in addition to ADH, a portion of alcohol would be metabolized by a pathway that is independent of ADH, likely by MEOS, which could account for the higher fraction ranging from 20% up to 25% of the alcohol metabolism in vivo [29]. The percentage contribution of MEOS in alcohol metabolism will be increased at higher alcohol levels, commonly achieved for instance, during social drinking, and it will adaptively be enhanced following chronic alcohol use. With respect to MEOS, its high *K*_m_ value for ethanol (Table 1) [26,27,38,42] favors the role of MEOS at higher alcohol concentrations [22,23,245]. MEOS inducibility by chronic alcohol use (Table 1) [26,27,30] underscores its importance in removing alcohol more quickly under conditions of preexisting and long lasting alcohol consumption, considering that MEOS may contribute >25% of overall hepatic alcohol metabolism [245,246,247]. It has also been suggested that when corrected for microsomal losses during preparation, half to two thirds of the increase in the rate of ethanol oxidation after chronic alcohol use can be accounted for by MEOS [29]. The existence of an ADH-independent pathway such as MEOS was confirmed by a variety of subsequent experimental studies and evaluations in humans [18,48,86,87,245], reinforcing its role at high alcohol concentrations and after chronic alcohol use [22,23,45,48,244,245,246,247,249,250,258,259,260,261,262,263,264]. 

**Figure 5:**
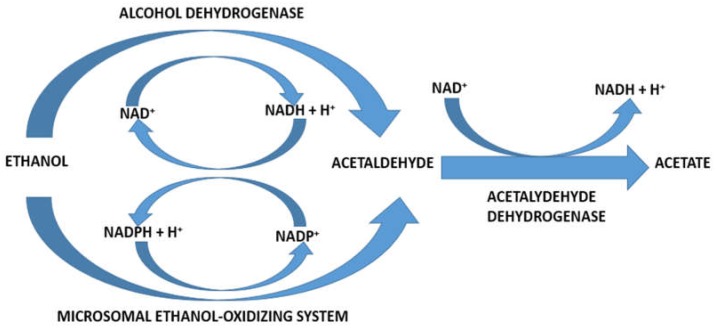
Interconnected action of hepatic alcohol dehydrogenase (ADH) and the microsomal ethanol-oxidizing system (MEOS). ADH produces reducing equivalents that are used by MEOS, showing that both enzymes depend on each other. The original figure was published in an earlier report [25], reproduced with permission of the Publisher Taylor & Francis (Didcot, UK).

Assessing the quantitative role of MEOS in alcohol metabolism is a particular issue, due to confounding factors such as the genetic isoenzyme variabilities of hepatic enzymes that are involved in hepatic alcohol metabolism. Other uncertainties relate to the extent of metabolic interactions between ADH and MEOS at the level of reducing equivalents produced by ADH and consumed by MEOS (Table 1). In more detail, MEOS and ADH may promote hepatic alcohol metabolism in joint action, because ethanol oxidation via ADH requires NAD^+^, and it generates with NADH + H^+^ reducing equivalents, which are welcome to MEOS, which requires reducing equivalents in form of NADPH + H^+^ (Figure 5). 

Due to the abundancy of publications retrieved from the database of PubMed and restriction of space and references, the selection will inevitably not cover all important aspects, but reports not references are not necessarily of lower quality. For a quick overview the following publications are listed (Table 3) [26,27,28,29,30,31,32,33,34,35,36,37,38,39,40,41,42,43,44,45,46,47,48,49,50,51,52,53,54,55,56,57,58,59,60,61,62,63,64,65,66,67,68,69,70,71,72,73,74,75,76,77,78,79,80,81,82,83,84,85,86,87,88,89,90,91,92,93,94,95,96,97,98,99,100,101,102,103,104,105,106,107,108,109,110,111,112,113,114,115,116,117,118,119,120,121,122,123,124,125,126,127,128,129,130,131,132,133,134,135,136,137,138,139,140,141,142,143,144,145,146,147,148,149,150,151,152,153,154,155,156,157,158,159,160,161,162,163,164,165,166,167,168,169,170,171,172,173,174,175,176,177,178,179,180,181,182,183,184,185,186,187,188,189,190,191,192,193,194,195,196,197,198,199,200,201,202,203,204,205,206,207,208,209,210,211,212,213,214,215,216,217,218,219,220,221,222,223,224,225,226,227,228,229,230,231,232,233,234,235,236,237,238,239,240,241,242,243]. 

## 8. Hepatic Acetaldehyde Dehydrogenase

Acetaldehyde generated via ADH and MEOS is further metabolized in the liver cell by mitochondrial acetaldehyde dehydrogenase (ALDH) to acetate (Table 1, Figure 5) [25], which is released into the bloodstream and oxidized to CO_2_ in various extrahepatic tissues. Little evidence is provided to show that the classic cytosolic aldehyde dehydrogenase in the liver contributes significantly to the metabolism of acetaldehyde [46,47]. Instead, two other enzymes exist, localized in liver mitochondria: one of these has a low affinity for acetaldehyde and is therefore less important, whereas the other one is described by a high affinity for acetaldehyde, requires NAD^+^ as cofactor, and is considered as the main pathway for acetaldehyde oxidation in the liver [46,47]. 

This high-affinity ALDH has been characterized in detail in rat liver mitochondria, which metabolizes acetaldehyde at a rate of 12 nmoles/min/mg of mitochondrial protein [47]. ALDH competes for NAD^+^ with cytosolic ADH, which also requires NAD^+^ (Table 1, Figure 5), and both enzymes generate NADH + H^+^, whereas MEOS requires reducing equivalents in form of NADPH + H^+^ (Table 1, Figure 5). 

**Figure 6:**
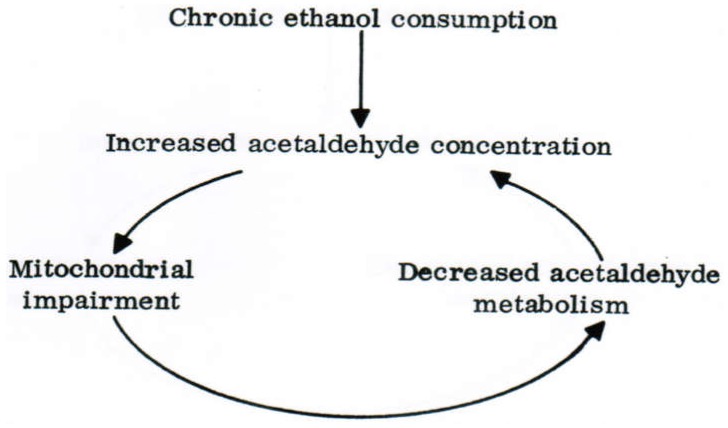
Hypothesis of a vicious circle of acetaldehyde in the liver. Acetaldehyde is increasingly generated from ethanol through MEOS, which is adaptively induced in activity following chronic ethanol consumption. Increased acetaldehyde levels in the liver in turn impair mitochondrial functions, including the activity of mitochondrial acetaldehyde dehydrogenase, which again likely enhances hepatic acetaldehyde concentrations at least temporarily, representing a vicious circle. Discussed and presented as a figure in a previous report [46], and reproduced with permission of the Publisher American Association for the Advancement of Science (AAAS, Washington, DC, USA).

This again shows the close interrelation between ALDH, ADH, and MEOS at the level of reducing equivalents. Of more importance, chronic alcohol consumption decreased acetaldehyde oxidation in intact mitochondria and reduced the ALDH activity in liver mitochondria disrupted by deoxycholate [46]. This reduction initiates a vicious circle due to an accumulation of acetaldehyde, which is toxic to the liver (Figure 6) [46]. 

**Figure 7:**
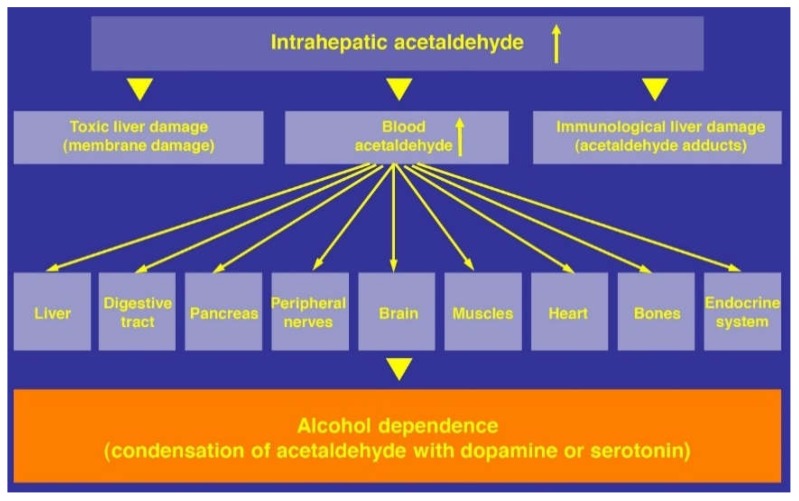
Actions of acetaldehyde. The increasingly generated acetaldehyde in the liver spills over in the blood and reaches many organs, which are injured by direct toxic attacks or through condensation products. Alcohol dependence is considered to be triggered by the condensation of acetaldehyde with dopamine or serotonin. Symbol ↑: Increase.

Through a condensation process with dopamine or serotonin, acetaldehyde facilitates the development of alcohol dependence [265] and can injury other organs including the brain. Higher blood acetaldehyde levels are found in alcoholic patients, as compared to non-alcoholic individuals [264], assuming a contributory role of the induced MEOS for the increased blood acetaldehyde levels due to an increased metabolism of ethanol to acetaldehyde in the liver. Sufficient circumstantial evidence exists that acetaldehyde is more injurious than ethanol (Figure 7).

Acetaldehyde plays a critical role in East Asian individuals, who are genetically deficient in hepatic ALDH2, which upon alcohol drinking causes increased blood acetaldehyde levels and the so-called clinical Asian facial flush [24]. These symptoms are also found in East Asians with a genetically more active form of liver ADH1B*2, converting ethanol to acetaldehyde in excess as compared to individuals lacking this super-active ADH variant [24]. With ADH1B*3, another super-active ADH variant is known, and found primarily in populations of African descent and Native Americans. Ethanol is metabolized through these two super-active ADH variants at a 30–40-fold increased rate to acetaldehyde, compared to normally functioning ADH enzymes encoded by the wild type ADH1B*1 gene. Clearly, individuals suffering from increased acetaldehyde levels due to such genetic variants of ALDH or ADH will abstain from alcohol drinking, preventing flush and the risk of alcohol dependence and alcoholic liver disease.

## 9. Cascade of Molecular Mechanisms and Cellular Events

### 9.1. The 5-Hit Working Hypothesis of ALD

**Figure 8:**
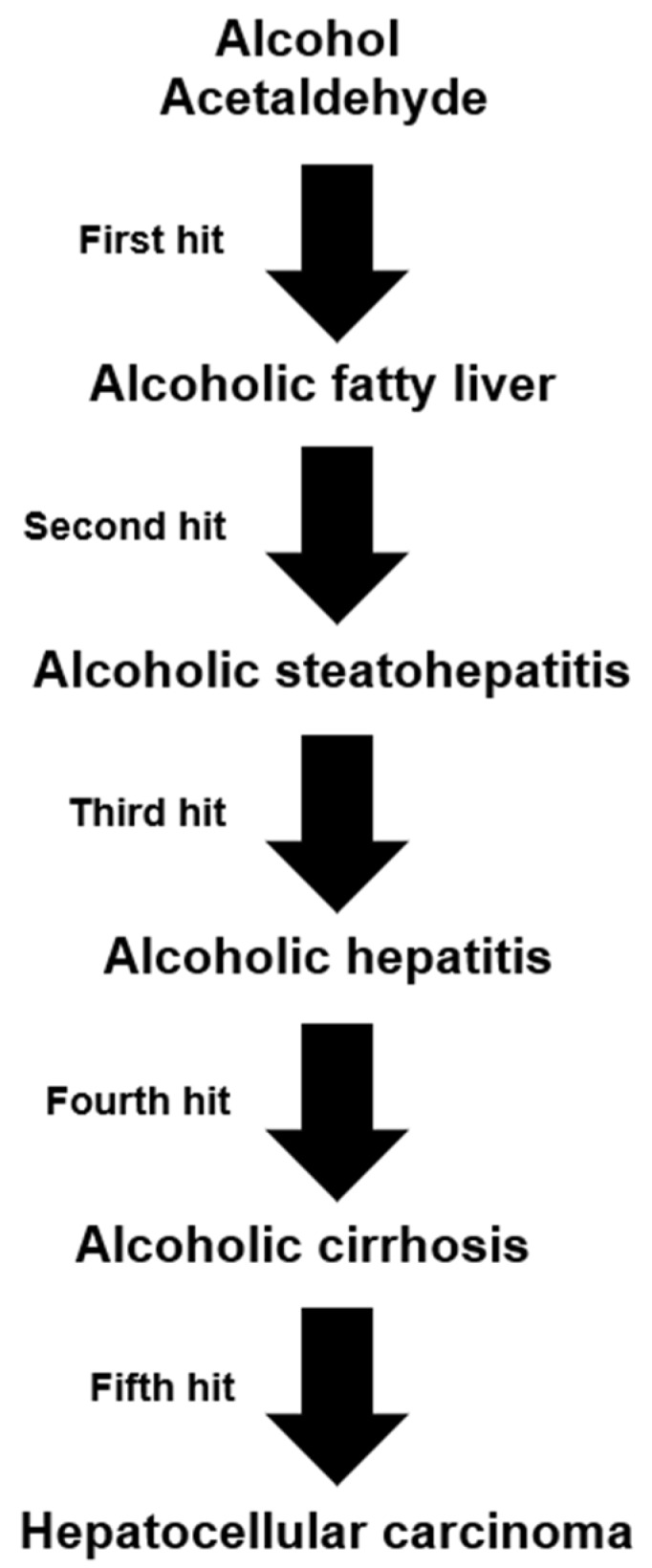
The 5-hit working hypothesis in alcoholic liver disease. The 5-hit hypothesis presents various possible steps leading from alcoholic fatty liver, eventually to hepatocellular carcinoma. In clinical practice, some patients with alcoholic hepatitis do not have steatosis/steatohepatitis as a precursor, with additional details provided in Table 4. The original figure was published in an earlier report [25] and is reproduced with permission of the Publisher Taylor & Francis (Didcot, UK).

ALD in patients with a history of prolonged alcohol abuse includes several stages. Among these are: alcoholic fatty liver (AFL), alcoholic steatohepatitis (ASH), alcoholic hepatitis (AH), alcoholic cirrhosis (AC), and alcoholic hepatocellular carcinoma (AHCC), this warrants discussing the various steps leading to AHCC as the end-stage disease of ALD, and considering a proposed cascade of events promoted along with five injury hits [25]. A multi-hit hypothesis is well applicable in chronic liver injury of ALD caused by prolonged use of alcohol [25,266], and suggests classifying ALD as a multi-hit disease. A hit hypothesis is also useful in idiosyncratic DILI due to use of conventional drugs, considering that one hit will follow the other [267]. The various stages of alcoholic liver disease are presented in connection with their respective hits (Figure 8).

The concept of a multi-hit disease as outlined for ALD (Figure 8) is under similar discussion also for a variety of other diseases including cancer, chronic disabling diseases, and more recently, obesity with nonalcoholic fatty liver disease (NAFLD) or nonalcoholic steatohepatitis (NASH) [115,137,138,207,209,268,269,270]. 

Of limited clinical value is such a multi-hit hypothesis for acute liver injury, caused for instance, by a single ingestion of overdosed acetaminophen that initiates intrinsic rather than idiosyncratic liver injury [271], or by acute intoxication of aliphatic halogenated hydrocarbons such as carbon tetrachloride, leading to acute toxic hepatitis [257,272].

The proposed 5-hit working hypothesis of ALD is based on highly complex and partially disputed conditions, thereby certainly open for critical discussion also regarding its potential clinical impact and whether the patient could benefit therapeutically from these considerations [22,25]. However, it clarifies several key factors leading to the five stages of ALD and outlines mechanistic sequelae as cascade of events (Table 4). 

### 9.2. Hepatocytes versus Non-Parenchymal Cells

**Figure 9:**
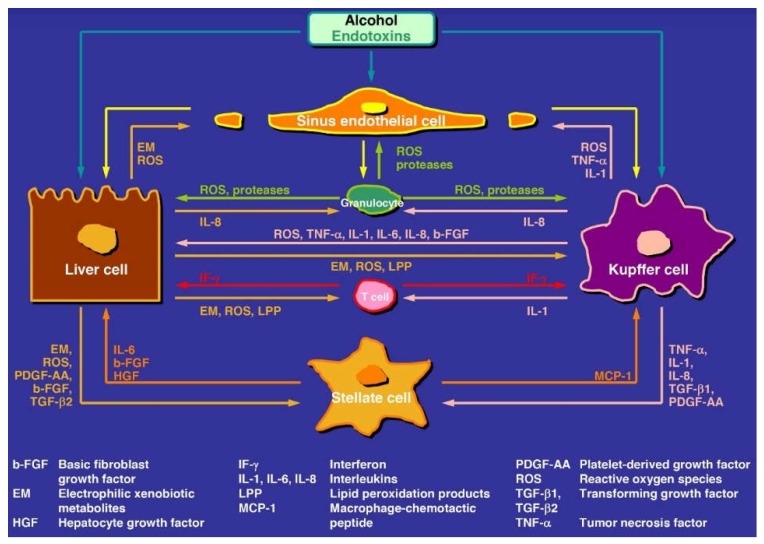
Hypothetical steps leading to alcoholic hepatitis. The pathogenesis of alcoholic hepatitis involves various mediators and cell types of the liver, some of the steps need confirmation and are therefore hypothetical. The original figure was published in a recent report [25] and is reproduced with permission of the Publisher Taylor & Francis (Didcot, UK).

Hepatocytes are involved in the development of ALD and govern the metabolism of ethanol and acetaldehyde in the liver [22,45,234,244,245,246,247,248,249,250,251,252]. For a quick overview, the complexity of ALD is illustrated as an example for AH, and it includes issues such as the involvement of hepatocytes and several non-parenchymal cells of the liver, the role of mediators, and the generation of multiple ROS (Figure 9).

In order to maintain the normal biologic and immunologic functions and regulate the homeostasis of the liver, the various cells are closely connected through mediators (Figure 9).

#### 9.2.1. Kupffer Cells

KCs contain CYP 2E1 similar to hepatocytes [195] and contribute to the pathogenesis of ALD through their activation by gut bacteria derived endotoxins and lipopolysaccharides, which are taken up by the liver in increased amounts [273]. As a result and likely due to specific actions of CYP 2E1 possibly upgraded following chronic alcohol consumption, activated KCs produce large amounts of ROS and mediators (Figure 9) and thereby promote ALD in joint actions with hepatocytes, HSCs, and LSECs, but details require further confirmatory studies. Although these events are suggestive as potential targets of pharmacotherapy in ALD [273], such specifically targeted therapy approaches were mostly disappointing with respect to severe AH [25] and these failures are best explained by additional pathogenetic mechanisms.

#### 9.2.2. Hepatic Stellate Cells

HSCs are known as key cells responsible for collagen synthesis, leading to alcoholic liver fibrosis and cirrhosis [274,275]. For this purpose, HSCs proliferate and undergo activation through phenotypic transdifferentiation into collagen-producing myofibroblasts [275]. The mechanism involved in this cell proliferation and activation was considered in response to necrosis and inflammation, partially triggered by mediators derived from KCs. However, the observation that in ALD, fibrosis can develop in the absence of AH, questioned the concept of necrosis an inflammation as obligatory factors of the proliferation and activation process. Instead, activation of HSCs was found to correlate with the severity of steatosis in a study cohort [275], requiring confirmation by other studies.

#### 9.2.3. Liver Sinusoidal Endothelial Cells

LSECs are continuously exposed to the blood of the liver sinusoids, since they form their walls [276]. They are known for their endocytic activity, which is high in well-functioning cells with intact fenestration, but impaired if cell fenestration is reduced [276]. In alcoholic liver injury, capillarization and lack of LSEC fenestration prevail, which is considered permissive for HSC activation and fibrosis under the guidance of various mediators.

### 9.3. Oxidative Stress and Reactive Oxygen Species

Along with oxidative stress, there is a continuous generation of radicals including ROS by not only hepatocytes but also non-parenchymal cells and leucocytes (Figure 9). Because of incomplete intracellular oxygen splitting, radicals are part of common life in any healthy organism such as plants [277,278,279,280], animals [280], and humans [281,282]. ROS and the other reactive radicals are commonly scavenged by antioxidants and thereby detoxified. Disease occurs if these protective mechanisms are lacking, with obesity as a good example because radicals play an important role for many obesity-related co-morbidities [281]. ROS is also implicated in liver injury [282,283] including alcoholic liver disease [92,231,240] where various forms of ROS and reactive radicals are contributory (Table 5).

Large amounts of ROS are produced in both subcellular areas, the mitochondria where ROS is injurious, and in the endoplasmic reticulum, where MEOS is primarily located. Fairly good evidence exists that ethanol oxidation via MEOS is promoted in some way by hydroxyl radicals [50,56,92,152,162] or superoxide radicals [56,152], leading to intermediates such as hydroxyethyl radicals [221]. Phospholipids are obligatory constituents of MEOS [30,49,57], likely in the form of lipid peroxides [205]. 

### 9.4. Signaling Mediators

Abundant signaling mediators are produced by hepatocytes and non-parenchymal cells, with details as examples being referenced in some selected reports [273,274,275,276,277]. Mediators leave the producing cells and modulate other cells; some of these signaling pathways are illustrated, and clearly classified as hypothetical steps, to be on the cautious side (Figure 9). It is outside of the focus of this article to provide additional references and a more thorough discussion on the individual mediators, since many suggested functions are yet debated.

## 10. Clinical Issues of Alcoholic Liver Disease

### 10.1. Natural Course

**Figure 10:**
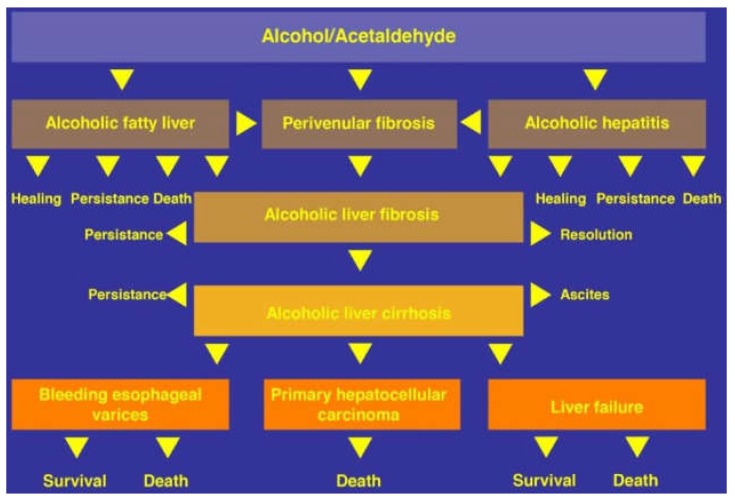
Stages of alcoholic liver diseases with potential clinical outcomes. The clinical outcome is variable among the different stages. Clinical deterioration is most commonly associated with continuation of alcohol use.

It is estimated that around 90–95% of the patients with a history of alcohol abuse will experience a fatty liver, whereas one third will advance to fibrosis and cirrhosis [6]. However, more robust data can only be achieved in a cohort of AFL with sequential liver biopsies, an approach that is clearly not acceptable, due to ethical reasons. The natural course of ALD is complex and complicated by serious, potentially life-threatening events (Figure 10).

### 10.2. Questionaires

**Figure 11:**
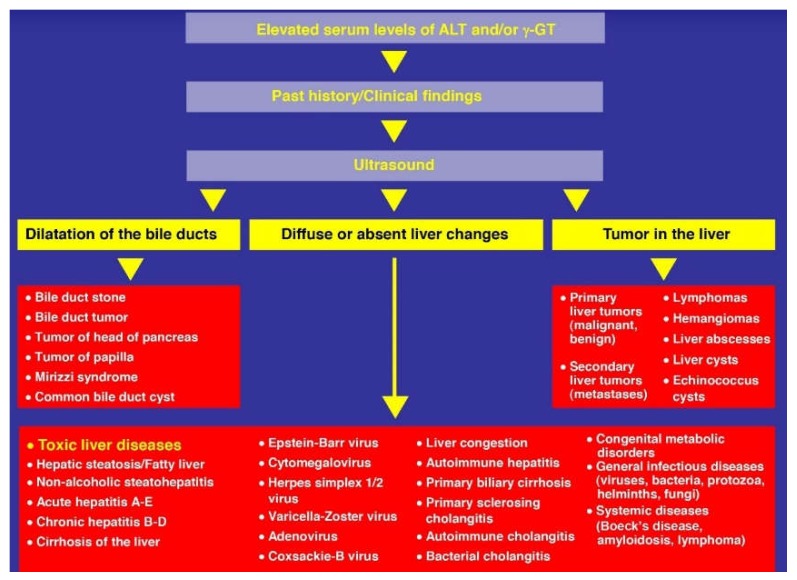
Differential diagnosis of alcoholic liver disease. Patients with a history of alcohol abuse presenting with increased liver values, require a careful diagnosis to exclude liver diseases that are unrelated to alcohol abuse.

Prerequisite for the diagnosis is among others that the alcohol abuse is confirmed by specific questionnaires, best to be evaluated by canonical tests [284,285,286,287], as recommended recently [25]. One of these is the CAGE questionnaire [284]. Alternatively, the more complex Michigan Alcoholism Screening Test (MAST) may be applied [285]. Both questionnaires are under discussion, regarding issues of validation, such as sensitivity and specificity [286,287]. These questionnaires facilitate not only the screening for alcoholism itself, but will also help exclude alcohol-unrelated liver diseases as confounding variables, using diagnostic parameters, as outlined in a previous publication [25]. Patients with ALD commonly use drugs and herbal products, making them susceptible for DILI [288,289] and HILI [290]. Their causality should be excluded or verified using the updated version of the RUCAM (Roussel Uclaf Causality Assessment Method) [291], considering that among patients with initially assumed DILI, almost 5% of them had not DILI but ALD [292]. Many other alternative diagnoses are to be excluded in patients with ALD to be sure that ALD is really ALD (Figure 11).

### 10.3. Laboratory Approaches

The search for individuals with severe alcohol abuse can be assisted by laboratory data with variable percentages of sensitivity: carbohydrate-deficient transferrin (CDT; 63%), gamma-glutamyltransferase (GGT; 58%), mean corpuscular volume of erythrocytes (MCV; 45%), aspartate aminotransferase (AST; 47%), alanine aminotransferase (ALT; 50%), and GGT + CDT (90%) [6]. However, and as expected, the specificity of these parameters is low. Known for a long time in clinical practice, high serum GGT activities in alcoholic patients created interest among hepatologists and led to experimental and clinical studies on its pathogenetic mechanisms.

In experimental studies on GGT activities due to alcohol feeding [293], using the liquid alcohol diet of Lieber and DeCarli [294], it was found that in animals, prolonged alcohol use reproduced the clinical findings of increased serum GGT activities in patients with alcohol abuse. Experimentally, chronic alcohol consumption led to statistically significant increases of GGT activities in the serum by 249% and in the liver by 60% [295]. This led to the conclusion that increased GGT activities in the serum are likely the result of an inducing effect of alcohol on liver GGT, and they cannot be ascribed alone to injurious properties of the alcohol, which would decrease the hepatic GGT activities due to enzyme release out of the liver cell. A similar increase of hepatic GGT activity was found in the liver of patients with ALF, associated with increased values in the serum [295]. Additional experimental studies showed that chronic alcohol use induced and doubled GGT activities in the microsomal fraction obtained after ultracentrifugation of liver homogenates [293]. Prolonged alcohol use increased GGT activities also in the liver plasma membranes, more so in bile canaliculi-enriched ones than in those free of bile canaliculi [296,297]. In other experimental studies, chronic alcohol consumption was found to increase bile flow, biliary GGT excretion, and bile acid output, all parameters that are assessed after cannulation of the bile duct [298]. The relationship between the biliary output of total bile acids and GGT was plotted against each other, and this showed an apparent linear relationship between both parameters in alcohol-fed animals, as well as in their pair-fed controls [298]. It seems that bile acids play a major role as natural chemicals solubilizing the GGT, an enzyme that is firmly bound to or tightly embedded in the liver plasma membranes and microsomal membranes. This view is supported by increased serum bile acid levels after experimental chronic alcohol use [298], in line with similar data obtained in patients with ALD [299]. 

**Figure 12:**
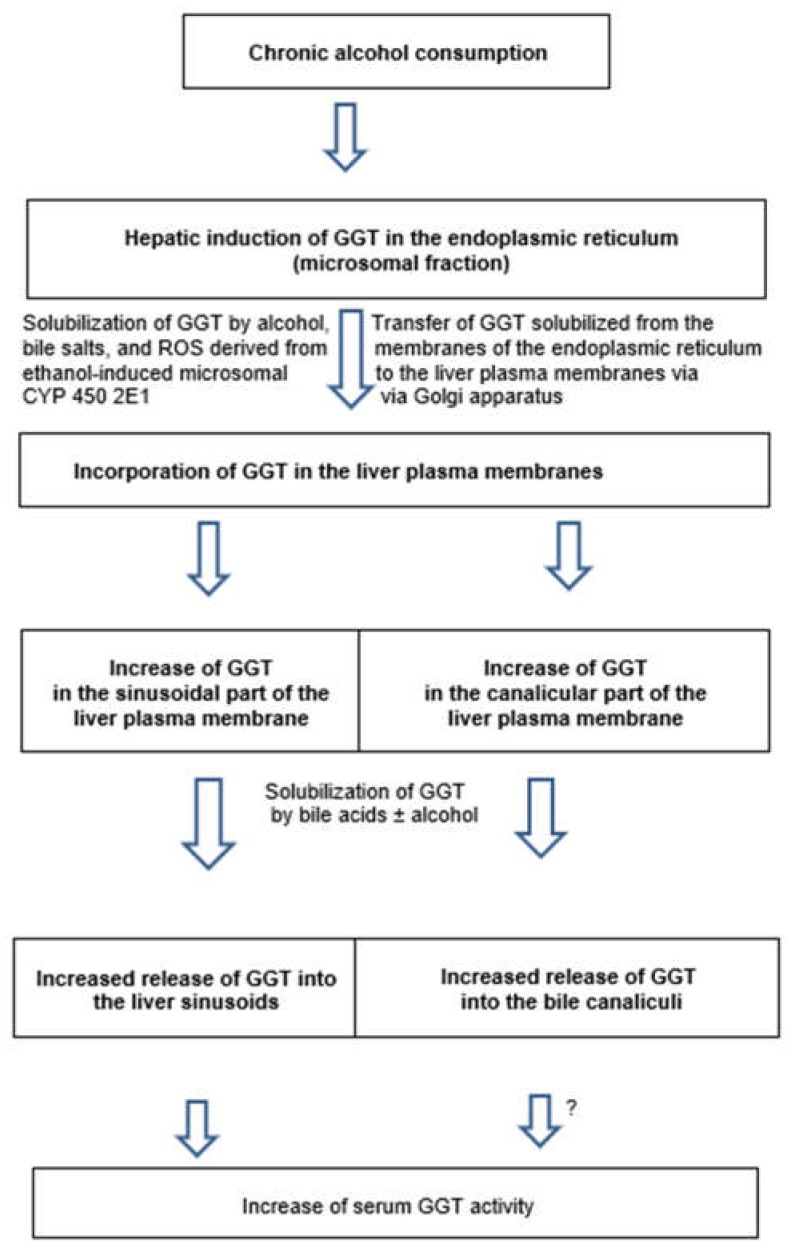
Hypothesis of events, leading to increased serum gamma-glutamyltransferase (GGT) activities, following chronic alcohol consumption. Mechanisms leading to increased GGT in the serum following alcohol abuse include microsomal GGT induction and enzyme solubilization via ethanol and bile acids. Symbol: ?, process under discussion. Abbreviation: GGT, gamma-glutamyltransferase.

Moreover, in vitro addition of deoxycholate caused increased GGT activities in liver microsomes of control and alcohol-fed animals, likely because of enzyme solubilization and its release out of the microsomal membrane [293]. Conversely, ethanol added in vitro to the microsomal fraction failed to enhance microsomal GGT activity of control animals, but increased enzyme activity in alcohol-fed animals, substantiating that chronic alcohol use may predispose to membrane GGT solubilization. In other experiments, the acute effect of alcohol on various constituents of the bile was evaluated, whereby rats received intravenously administered 0.9% NaCl solution alone or containing in addition ethanol [300]. Compared to the control group receiving saline alone, ethanol infusion significantly increased biliary gamma-glutamyltransferase excretion by 166% (*p* < 0.0125); while bile flow and biliary excretion of both total bile acids and alkaline phosphatase remained virtually unchanged. The selective increased biliary excretion of gamma-glutamyltransferase through the action of ethanol is best explained by an augmented solubilization of GGT, an enzyme found in the small bile canaliculi of the liver cells and the epithelial cells of the larger bile ducts. Alcohol infusion failed to change the excretion of total bile acids via the bile, suggesting that the selective solubilization of gamma-glutamyltransferase occurs by a mechanism primarily not involving total bile acids. Instead, it is likely caused by alcohol with its physico-chemical properties that may lead to the increased fluidity of liver plasma membranes [300]. Some aspects of mechanisms leading to alcohol-related GGT increase in the serum have been summarized (Figure 12) [301]. 

**Figure 13:**
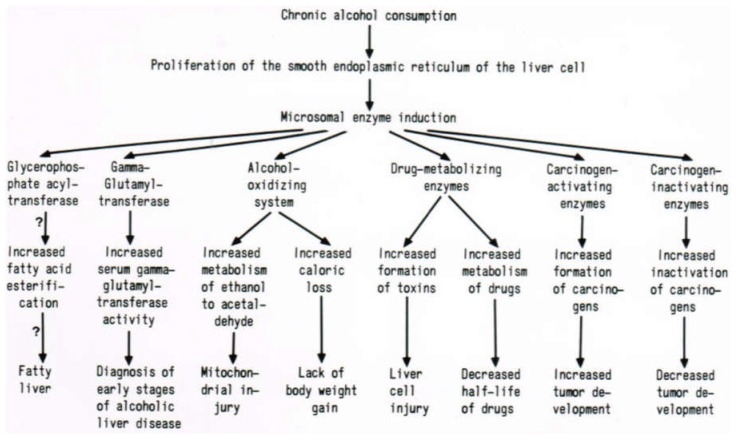
Proliferation of the smooth endoplasmic reticulum associated with microsomal induction of gamma-glutamyltransferase (GGT) due to alcohol abuse. Chronic alcohol consumption induces also various other microsomal functions, which are of potential clinical relevance. In addition, increased GGT activities of the plasma membranes may contribute to increases in the serum [296,297]. Symbol: ?, under discussion. The original figure was published in an earlier report [23] and is reproduced with the permission of the Publisher Wiley (Hoboken, NJ, USA).

Chronic alcohol consumption causes induction of GGT and various other parameters in the endoplasmic reticulum of the hepatocyte [23], with clinical implications (Figure 13). 

In essence, chronic alcohol consumption causes induction of GGT in the endoplasmic reticulum of the hepatocyte (Figure 13) [23], from which GGT is solubilized by alcohol, bile acids, and ROS derived from ethanol-induced CYP 2E1, tightly associated with GGT in the microsomal membrane, is translocated via the Golgi apparatus to and incorporated in the liver plasma membranes (Figure 12). These membranes may be susceptible for partial solubilization of GGT through the action of alcohol and increased bile acid levels, a process that facilitates spilling over of the solubilized GGT into the blood of the liver sinusoids, leading to increased GGT activities in the blood of the systemic circulation (Figure 12 and Figure 13). Here, its activity can then be assayed in the serum of patients.

**Figure 14:**
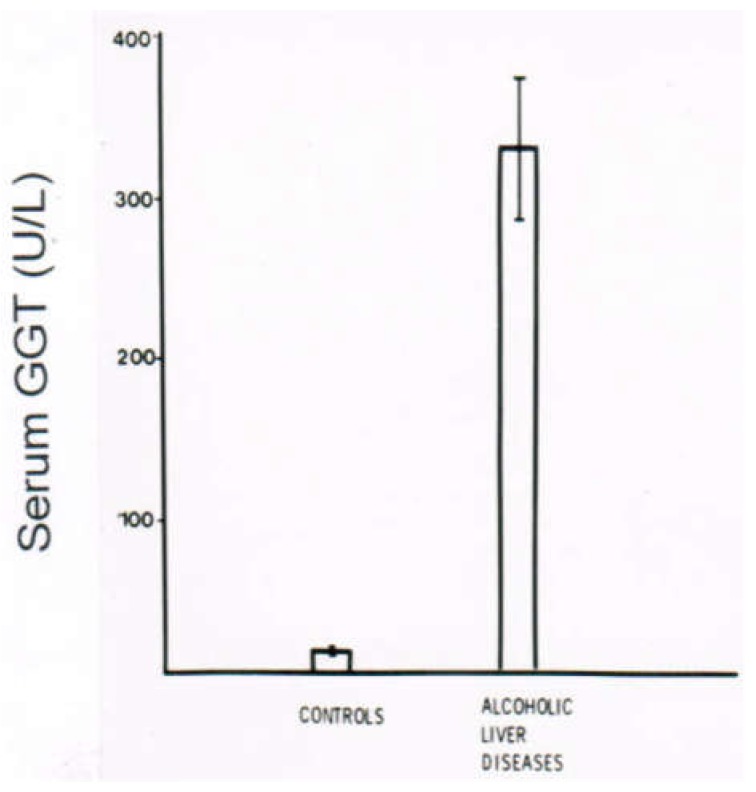
Serum gamma-glutamyltransferase (GGT) activity in alcoholic liver diseases. Patients with an alcoholic liver disease show increased serum GGT activities as compared to a control group lacking a previous history of alcohol abuse and with normal liver tests.

**Figure 15:**
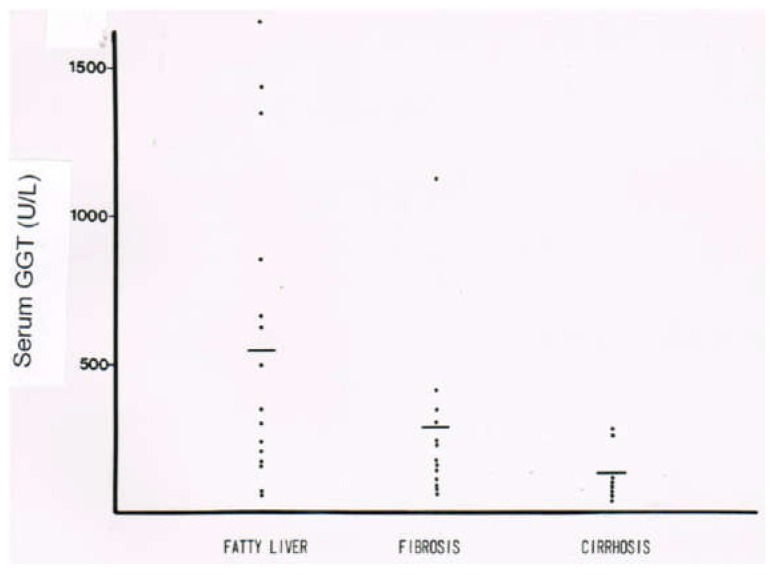
Serum gamma-glutamyltransferase (GGT) activities in patients with different stages of alcoholic liver diseases. Highest GGT activities were found in patients with alcoholic fatty liver, with decreasing values along with increasing fibrosis. Relative low values are found in patients with alcoholic cirrhosis, possibly due to reduced GGT enzyme induction because of impaired liver function.

**Figure 16:**
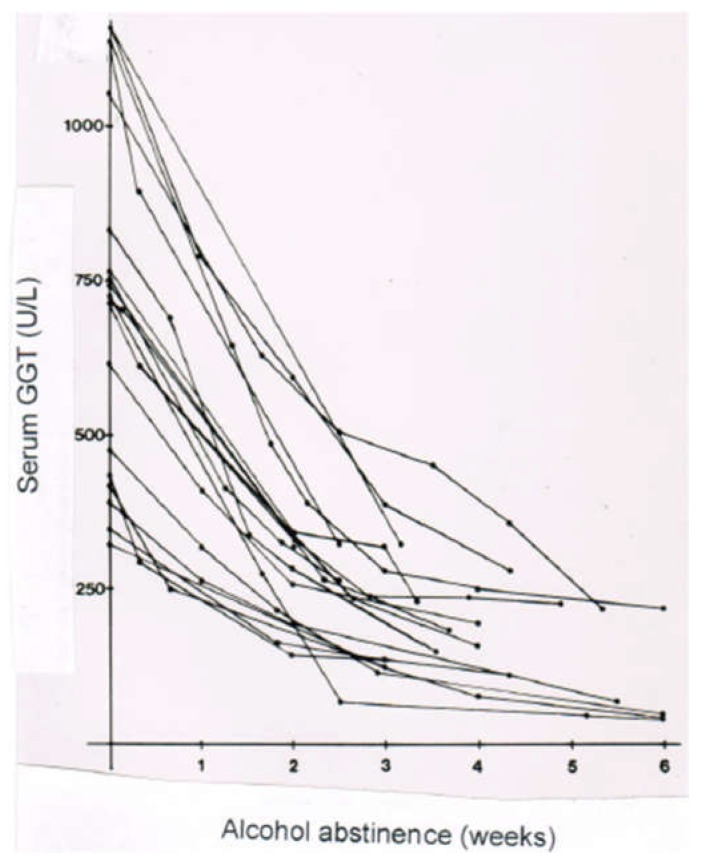
Decline of serum gamma-glutamyltransferase (GGT) activities due to alcohol abstinence. Alcohol abstinence leads to a reduction of serum activities of GGT in patients with alcoholic liver disease of all stages including alcoholic fatty liver, alcoholic steatohepatitis, alcoholic hepatitis, and alcoholic cirrhosis. This approach is extremely valuable in any clinical setting for checking whether a patient has followed the professional advice to stop alcohol use. The original figure was published in a previous report [301] and is reproduced with the permission of the Publisher Springer (Berlin, Germany).

In a clinical setting of patients with ALD, serum activities of GGT are high, as compared to a control group lacking a history of alcohol abuse and presenting with normal GGT values (Figure 14). The highest serum GGT activities are found in patients with AFL, with a decreasing tendency, along with increasing fibrotic changes and transition of fibrosis to cirrhosis (Figure 15). These results were obtained in a German population, and they differ substantially from a recent study in Beijing that showed no differences of serum GGT activities among the various stages of ALD [302]. Abstinence from alcohol use commonly leads to a decline of serum GGT activity, a useful approach for detecting relapse of alcohol use (Figure 16) [301].

Patients with alcoholic fatty liver show mostly striking increased GGT activities in the serum, associated with corresponding increases in the liver; these data were reproducible in an animal model of chronic alcohol feeding (Table 6) [295], useful for studying the mechanism of increased serum GGT activities [293,296,297,298,300].

Serum ALT and AST activities are often rather low in ALD [295], including AFL (Table 7). 

The serum ratio of AST/ALT provided diagnostic clues in ALD [303]. The ratio is significantly increased in patients with alcoholic hepatitis and cirrhosis (2.85 ± 0.2), and much higher compared to cirrhosis of the so-called postnecrotic type (1.74 ± 0.2), not further specified chronic hepatitis (1.3 ± 0.17), jaundice due to biliary obstruction (0.81 ± 0.06), and not further specified viral hepatitis (0.74 ± 0.07). This led to the proposal that a serum AST/ALT ratio >2.0 is highly suggestive of alcoholic hepatitis and cirrhosis [304], found in as much as 70% of this special cohort, but in only 26% of postnecrotic cirrhosis, 8% of not further specified chronic hepatitis, 4% of acute viral hepatitis without specification, and none of jaundice caused by biliary obstruction. Similar data were published in a study from China, which confirmed a striking increase of the AST/ALT ratio for severe AH (2.08 ± 1.23), but a lesser one for AC (1.44 ± 0.96), whereas the ratio was lower in mild AH (0.66 ± 0.72) and AFL (0.62 ± 0.51) [302]. In Germany, the values of the AST/ALT ratio have been published for patients with AFL (1.64 ± 1.57), as compared with a corresponding control group consisting of individuals with normal liver histology and normal values of AST, ALT, and GGT; this showed a lower AST/ALT ratio (0.72 ± 0.24) that did not achieve a statistically significant level, due to the small control group, and the variability of results within the AFL cohort [295]. Data variability appears indeed to be a major problem in patients with established alcohol abuse and AFL (Table 7). Nevertheless, an AST/ALT ratio of >1.0 can help identify individuals with problematic alcohol use without overt liver alcoholic liver disease, while a ratio <1.0 cannot exclude alcohol abuse with the required d level of certainty. Of interest in the clinical context, serum glutamate dehydrogenase (GDH) activity may be a good marker for alcoholism, with the preference of liver cell necrosis in the alcoholic, showing virtually no overlap between patients with and without AH [305]. Serum GDH activity was also found, with a 5-fold increase in patients with AFL, which was significantly different from controls [304] and showed little variability (Table 7).

With the toxic acetaldehyde as the first oxidation product derived from ethanol, it is not unexpected that antibodies against acetaldehyde proteins are generated and found in the serum of individuals with an alcohol abuse and an obligatory alcohol metabolism. Such serum antibodies can be determined in patients with AH, but their diagnostic validity in a clinical setting is unclear [304]. In this diagnostic context, there are suggestions that blood microRNAs could be of value as diagnostic biomarkers in AH [306], but this issue is yet debated for other liver injuries including DILI or HILI [307,308].

## 11. Alcoholic Fatty Liver

**Figure 17:**
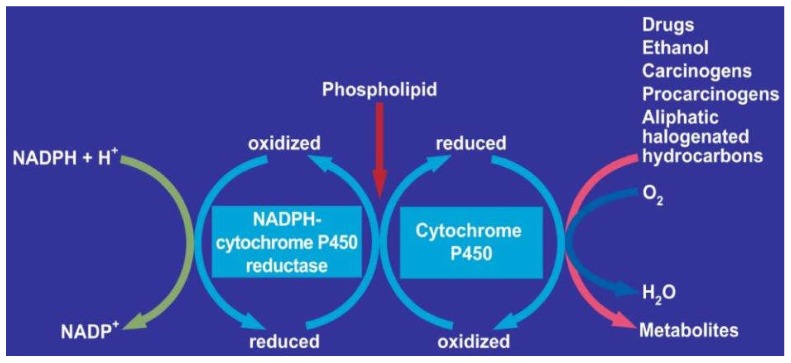
Risk factors of exogenous substrates for alcoholic fatty liver. At the stage of alcoholic fatty liver, and due to microsomal induction of cytochrome P450, various exogenous substrates are increasingly metabolized, leading to additional liver injury or to decreased blood drug levels.

AFL is a liver injury that is completely reversible upon abstinence from alcohol [22], and it lacks a lethal course during hospitalization [302] except in rare, extremely severe cases [22]. Early recognition is mandatory to advice stopping alcohol use and to prevent more serious stages of ALD. Symptoms are not specific and rarely described [4,5,6,9]. By convention, diagnosis of AFL requires a fat deposition >5% of the liver cells, and presents mostly as macrovesicular steatosis, and rarely as the microvesicular type [309]. Clinical differentiation of AFL is mandatory especially from NAFLD and NASH (Figure 13), commonly associated with obesity and its comorbidities [270,281,310]. Patients with AFL are prone to injurious hits by various drugs and chemicals including carbon tetrachloride [39,257,272], with augmented risk due to increased metabolism related to the induction of CYP 2E1 (Figure 17). 

## 12. Alcoholic Steatohepatitis and Alcoholic Hepatitis

Reports often do not distinguish between ASH and AH, but a clear differentiation is essential for reasons of clarity to describe their characteristic features of the natural clinical course and treatment options [25]. Unlike ASH, AH commonly presents as a severe disease with a high risk of complications. Similar to AFL, clinical symptoms are rarely observed in patients with ASH, whereas AH is characterized by poor nutritional condition, enlarged liver and spleen, jaundice, ascites, mental alterations, and hepatic as well as renal insufficiency [311].

The liver histology of ASH is described as hepatic injury with steatosis [309,312,313,314,315]; characteristic are ballooned liver cells [313], lytic necrosis is more frequent than apoptosis [313,316]. Not pathognomic are Councilman bodies [313] and Mallory-Denk bodies [309,313]. Lobular inflammation mostly consists of neutrophils, commonly found nearby to ballooned hepatocytes [313]. The extent of histological changes determines the lethality risk of AH [311].

AH is commonly a type of acute-on-chronic liver disease [25]. Liver biopsy is certainly indicated under the assumption that the patient would benefit from the potential results of this invasive procedure, such as if pharmacotherapy options are to be discussed in severe AH [317,318]. 

Restricted to severe AH, pharmacotherapy options have been thoroughly reviewed [25]. In short, corticosteroids are most promising as the therapy of first choice [25], considering the results of clinical trials [319,320,321,322,323,324,325,326,327,328,329,330]. Pentoxifylline is recommended as a second-line therapy [331,332]. Preferentially indicated in patients with severe AH and concomitant infections or sepsis is pentoxifylline, not allowing steroid treatment [25]. Most importantly, patients not responding to a seven-day corticosteroid therapy should be taken off this treatment and then evaluated for liver transplantation, because other pharmacotherapy options are not available. This also rules out the use of pentoxifylline as rescue therapy because of missing efficacy in this particular cohort subset. A lack of efficiency in patients with severe AH was also provided for drugs like infliximab [333,334,335], propylthiouracil, N-acetylcysteine, silymarin, colchicine, insulin and glucagon, oxandrolone, testosterone, and polyunsaturated lecithin [25].

Despite pharmacotherapy options, the basic approaches of therapy and prevention remain, as are nutritional support and alcohol abstinence [25]. Malnutrition causing nutritional deficiencies due to impaired food and caloric intake is frequently observed [8]. For clinically relevant nutritional deficits that are associated with severe AH, published guidelines should be followed, to correct these conditions [9]. Alcohol abstinence is mandatory in all patients. For those patients with withdrawal risks, the use of Acamprosate (acetylhomotaurine) is advised [9]. 

**Figure 18:**
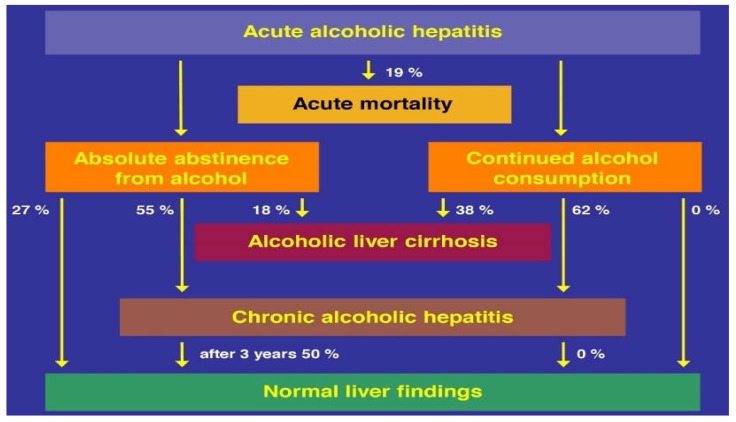
Natural course of alcoholic hepatitis under absolute alcohol abstinence or continued alcohol use. Data are compiled from results published in a previous report [336].

Although commonly recommended in various publications [8,9,336], alcohol abstinence does not necessarily prevent short-term hospital lethality in patients that are likely abstinent under hospital surveillance, with lethality rates from 15% [8,311] up to 60% [311]. In such clinical settings of obligatory alcohol abstinence, most patients with AH will likely improve or at least stabilize [336]. Absolute alcohol abstinence leads back to a normal liver (27%), but it eventually progresses to liver cirrhosis (18%), or leads to chronic alcoholic hepatitis (56%); after three years of continued alcohol abstinence, half of these patients reach a normal liver. The conditions are different for those AH patients, who maintain the consumption of alcohol use, because they will develop cirrhosis at a higher rate (38%) or chronic alcoholic hepatitis (62%) (Figure 18) [336]. 

Clearly, absolute alcohol abstinence must be the primary goal, not substitutable by reduced alcohol consumption (Figure 18). Many more clinical details can be retrieved from publications referenced recently [25], especially regarding guidelines issued by the AASLD (American Association of the Study of Liver Diseases) [9] and the EASL (European Association of the Study of the Liver) [8]. To evaluate patients with severe AH as potential candidates for liver transplantation, the following diagnostic instruments must be applied [7,8]: Maddrey score (Maddrey discriminant function); GAHS, Glasgow Alcoholic Hepatitis Score; ABIC score (that considers age, serum bilirubin, INR, and serum creatinine, leading to the acronym ABIC); and MELD score (Model-for-End-Stage-Liver-Disease) [25].

Liver transplantation is an option for selected patients with severe AH that are unresponsive to pharmacotherapy [25], but criteria of indications are yet disputed; they vary from country to country, and from continent to continent [8,9,10,11,12,13]. At least some consensus exists that the number of AH patients eligible for liver transplantation will remain low if the strict inclusion criteria for the waiting lists are followed and if donated organs are limited. The AASLD guidelines of 2010 are more cautious, considering recommendations by the American Society of Transplant Physicians and the AASLD, which had been published in 1998, and generally excluded AH patients from liver transplantation [5]. As opposed to AASLD, the EASL guidelines of 2010 mention the proposals of European and North American experts, who previously excluded AH as an indication for liver transplantation, reiterating that this has recently been challenged by a multi-center, case controlled study from France, which provided clear evidence of a higher survival rate in patients who benefitted from early liver transplantation.

Recent developments up to 2017 are summarized in four critical reviews, published on liver transplantation in AH [10,11,12,13]. In the first PRO transplantation article, Toniutto et al. from Italy favored early liver transplantation in a few selected patients with severe AH [10], in line with suggestions published in another PRO transplantation article by Artru et al. from France [11]. The third review was published from the US authored by Luce, who calls for a strong selection procedure, but otherwise titles his paper also as a PRO transplantation view [12]. Finally, the fourth review authored by Fung from Hong Kong was presented as a CON transplantation article [13]. For 2018, no actual guidelines of AASLD or EASL are to be expected, that would clarify the situation by providing a consensus paper on this issue.

## 13. Alcoholic Cirrhosis

Patients with AC often have no symptoms that would allow for diagnosis and in-time recommendations to abstain from alcohol use. Indeed, the diagnosis of AC would not be established unless until liver functions are heavily disturbed, causing symptoms, which is a common clinical experience. At hospital admission, when the diagnosis of alcoholic cirrhosis was established, in 24% of the patients, complications were not registered, 55% had only ascites, 6% experienced only bleeding from ruptured esophageal varices alone, 4% had variceal bleeding combined with ascites, and 11% had mental problems due to hepatic encephalopathy [337]. Likely due to the initially silent character of the disease, only about one third of the patients with alcoholic cirrhosis had been treated as in-patients before decompensation [8,338,339], a common experience among clinical hepatologists. However, the diseases progresses, and within the first year after admission, these patients are at increased risks of experiencing complications of liver decompensation. For instance, they face a 20% risk of developing ascites, a 6% risk of acute bleeding from ruptured esophageal varices, and a 4% risk of mental disturbances due to hepatic encephalopathy [8,337,339]. Early recognizable under professional health care surveillance, ascites are typically seen as the first complication [8], but other complications such as icterus, acute bleeding from ruptured esophageal varices, and mental disturbances caused by hepatic encephalopathy may also follow within a short period of time [8,337,339].

**Figure 19:**
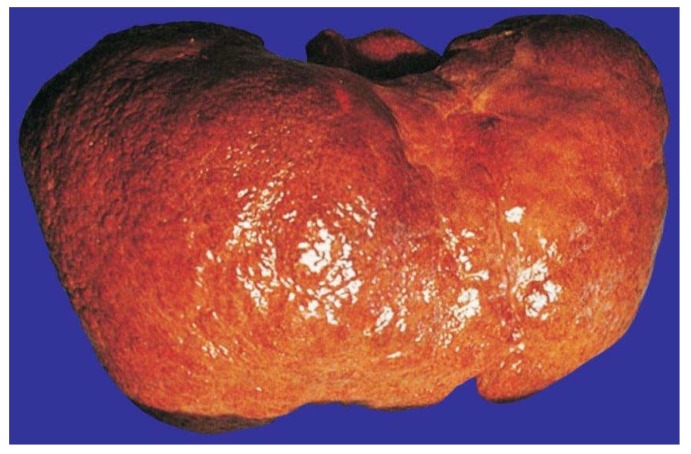
Macroscopic picture of alcoholic cirrhosis. The surface of alcoholic cirrhosis is granular, reflecting the regenerative nodules, which can be seen upon histological evaluation.

By liver histology, AC presents with thick collagen strands around the central vein and coursing through the hepatic lobules, also involving the perisinusoidal and pericellular areas [309]. In other areas, focal regeneration predominates, leading to regenerative cirrhotic nodules, mostly of the micronodular type. Regenerating nodules are clearly visible macroscopically on the liver surface with fragmented light reflexes (Figure 19).

**Figure 20:**
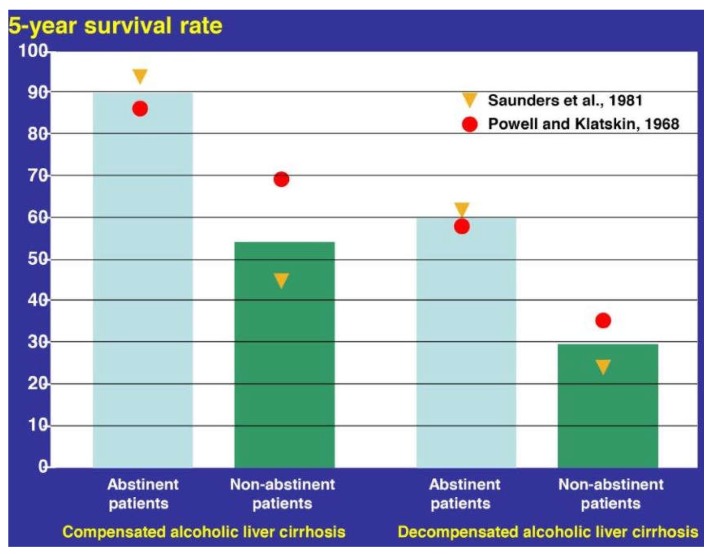
Prognosis of alcoholic cirrhosis. Shown is the 5-year survival rate, prognosis is better in abstinent patients with compensated cirrhosis as compared to abstinent patients with decompensated cirrhosis. In both cohorts, continued alcohol use deteriorates the survival rate. The original figure was published in a previous report [341] and is reproduced with permission of the Publisher Wiley (Hoboken, NJ, USA).

The pharmacotherapy of AC is disappointing. Among the specific therapies evaluated in clinical trials of patients with alcoholic cirrhosis, no benefit of outcome was achieved with S-adenosyl-l-methionine (SAMe), propylthiouracil, colchicine, anabolic-androgenic steroids, and silymarin [8]. Current management of alcoholic cirrhosis focuses on correcting nutritional deficiencies and alcohol abstinence, which substantially improves the 5-year survival rate in patients with compensated AC as well as decompensated AC (Figure 20) [338,340,341].

Liver transplantation (LT) is principally the only chance for some selected patients with decompensated AC to escape the potential fatal outcome, but the number of organs available for LT is limited, and alternatively using split livers donated by family members is not a good approach, due to some mental reservations [8,9,10]. However, LTs are also under general discussion and basic considerations as to whether patients with a history of alcohol abuse should be candidates for a LT [8,9,10]. Although the AC-related lethality rate of 1.36% is low during the hospital stay [302], abstinent patients with decompensated AC have a 5-year survival chance of only around 60%, whereas 40% of this cohort will die (Figure 20) [338,339,341]. Clearly, patients with compensated AC are not candidates for a LT, because of their excellent prognosis with a 90% chance of survival during the next five years, provided that they abstain from alcohol use and adhere to long-term abstinence with professional support; starting and maintaining abstinence is crucial, since non-abstinent patients have a 5-year survival chance of only 45–60% (Figure 20).

For inclusion in the LT waiting list, only a few patients with decompensated AC will be eligible [8,9,10], although at this stage, the 5-year survival rate is low, even in abstinent patients, but more so in non-abstinent ones (Figure 20). Despite the high prevalence of decompensated AC in Western countries, 95% of these patients were never formally assessed for their candidacy for LT [342]. The appropriate patients should be evaluated for LT in a similar way as other patients with decompensated liver disease are assessed, focusing on a careful evaluation of their medical background and psychosocial condition [9]. To achieve a complete assessment, a formal evaluation of the likelihood of long-term abstinence is helpful, considering that a 6-month period of abstinence has been recommended by most centers as a minimal listing criterion [8,9,10]. This period allows issues related to alcohol abuse to be addressed in patients with recent problems of alcohol use, but more importantly, it enables sufficient improvement of the clinical condition prior to the planned liver transplantation, or even better, makes LT unnecessary [9]. Controversial discussions focus on the utility of the 6-month rule as a predictor of long-term sobriety, and additional information on details of other stricter criteria for LT has been published [8,9,10]. Meeting most of these prerequisites, the patient survival rate at five years after LT was 73% [343], and thereby better than the cohort of decompensated AC without LT, although transplanted patients are highly likely to drink after transplantation, but this obviously has no serious effect on the transplanted liver and the overall clinical course [8].

**Figure 21:**
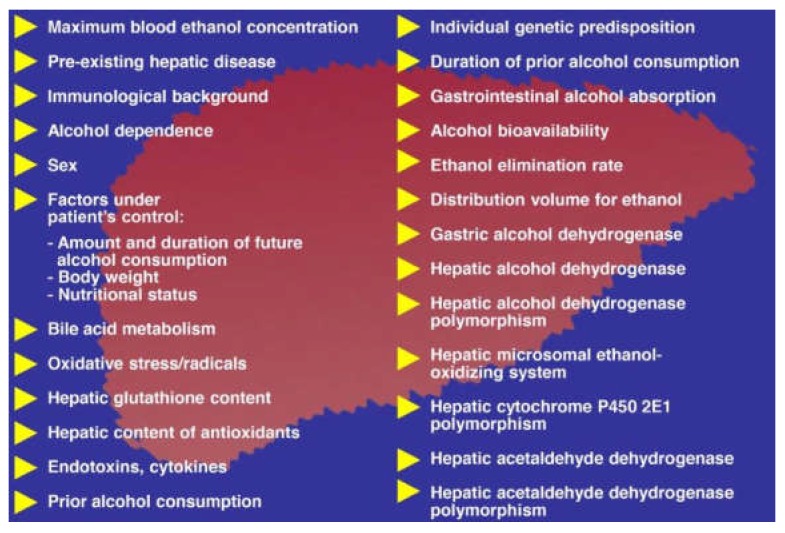
Hypothesis of risk factors of alcoholic cirrhosis.

Many risk factors of AC are well established, based on clear evidence, others are based on results that are controversially discussed [9]; some of the risk factors are listed (Figure 21). 

**Figure 22:**
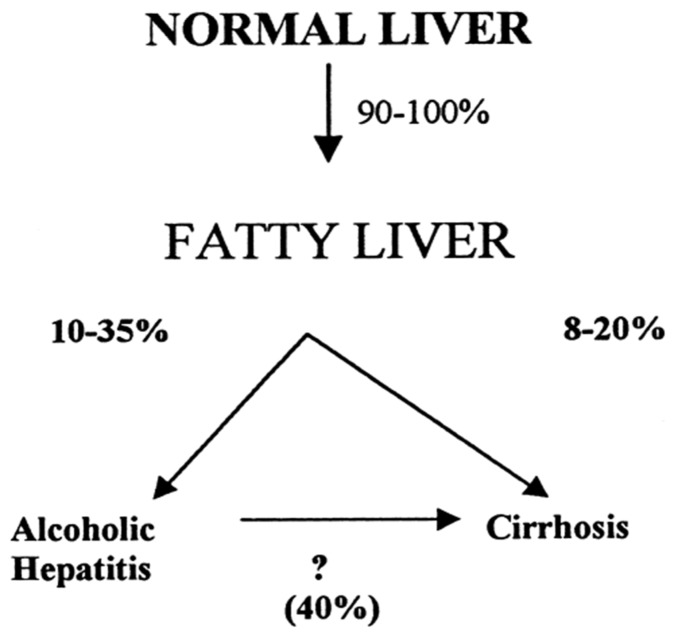
Development of alcoholic cirrhosis Good evidence exists that alcoholic hepatitis is responsible for most cases of alcoholic cirrhosis, but it may emerge also from alcoholic fatty liver with its perivenular and perisinusoidal fibrosis. Symbol: ?, pathway under discussion. The figure was published in a previous report [345], and is reproduced with permission of the Publisher Springer (Berlin, Germany).

Abundant factors determine whether cirrhosis develops or not. Most of these cannot be influenced by individuals who drink alcohol, but some factors are under patients’ control, to minimize the risk of upcoming liver injury. Avoidable risk factors include the amount and duration of future alcohol consumption, obesity, and poor nutritional status. It also should be recognized that the development of AC mostly occurs silently. Patients in whom ALF has been diagnosed should abstain from alcohol use, since occasionally fibrosis and cirrhosis starts as central hyaline sclerosis syn. perivenular fibrosis at the stage of AFL, without the clinical warning symptoms of AH (Figure 10) [9,309], an interesting proposal of early diagnostic relevance [344]. Based on several reports [5,6,8,9,345], a percentage sequelae leading to AC is proposed (Figure 22). 

Indeed, good clinical evidence is provided that fibrosis can occur in the absence of alcoholic hepatitis. This raises the question as to whether cell necrosis and inflammation are obligatory factors for alcohol-related hepatic stellate cell proliferation and activation and subsequent fibrogenesis and development of cirrhosis.

## 14. Alcoholic Hepatocellular Carcinoma

Patients with AC are at risk of AHCC, a rare complication of advanced ALD [346,347], but it can also can occur in alcoholic patients without AC [348]. During the development of AHCC, symptoms such as fatigue, weight loss, and anorexia increase in severity. To early recognize AHCC, patients with AC should have regular surveillance with ultrasound and the serum tumor marker α_1_-fetoprotein. Small localized AHCC can be treated by percutaneous ethanol injections; radiofrequency, microwave ablation, and liver segment resection are the ideal treatments [349], whereas for multifocal AHCC, only systemic chemotherapy or LT remains as a last option, disputed due to the problems of the final outcome [346,348]. The mechanisms leading to AHCC have not clearly been identified, but tumor initiation is likely triggered by ROS or acetaldehyde [346,347]. Experimentally, chronic alcohol consumption leading to AFL actually decreases tumor incidence by dimethylnitrosamine [83] and is protective for liver injury by dimethylnitrosamine [64,85], which is metabolized by CYP 2E1 [95,105]. Whether another carcinogen or procarcinogen may trigger AHCC is not known.

## 15. Actual Issues and Future Perspectives

A satisfactory unifying mechanism for individual susceptibility, initiation, and progression of alcoholic liver injury is not available [348]. Instead, some dozens of signaling mediators and mechanistic pathways have been presented, including CYP 2E1 and other topics, which are still challenging issues in experimental and clinical alcoholic liver injury, as results are in part contradictory, rarely confirmed, or discussed by other groups [349,350,351,352,353,354,355,356,357,358,359,360,361,362,363,364,365,366,367,368,369,370,371,372,373,374]. Much emphasis has previously been placed on the role of the endoplasmic reticulum as the principal localization of CYP 2E1, and the clinical and pathogenetic importance of this microsomal CYP 2E1, which is considered to play an essential role in endoplasmic reticulum (ER) stress syn. microsomal stress, thereby promoting liver injury [231,232,233,234,235,240,247,249,251,252], likely involving membrane lipid peroxides [348]. However, there is now growing evidence that this isoenzyme is also found in the mitochondrial compartment of the hepatocyte [351,352,353,356], an observation that raises a number of questions regarding the characteristics of this mitochondrial CYP 2E1, as compared to the previously well described microsomal variety [353,355]. In order to study this, special analytical techniques are required and were used, because conventional methods using ultracentrifugation to isolate liver mitochondria from liver homogenates are risky, due to possible contamination of the isolated intact mitochondria by microsomal fragments [353,356]. Among the preferred methods are those with focus on the proteasome complex and knockout and transgenic models [356,360]. More specifically, CYP 2E1 knockout models and transgenic models have been found to be valuable in describing a major functional role of CYP 2E1, as summarized and referenced in detail recently [358]. Using these CYP 2E1 knockout mice, a major role of CYP 2E1 was shown in experimental alcoholic fatty liver. In addition, overexpressed CYP 2E1 was found in a CYP 2E1 transgenic mouse model containing human CYP 2E1, which allowed for studies on the properties of human CYP 2E1, and provided corresponding validation. In these humanized CYP 2E1 knock-in mice, human CYP 2E1 transgene was introduced in the corresponding CYP 2E1 null mouse background [358]. Of some clinical interest are new results published on CYP 2E1 exosomes as possible diagnostic biomarkers in the blood of humans with alcoholism and microsomal stress [360]. They could help assign alcohol as a cause in a liver disease of unknown etiology, but in a clinical setting, other causes commonly have to be excluded, just to be on the safe side [25].

Notably, mitochondrial CYP 2E1 is induced by alcohol, as is microsomal CYP 2E1 [353]. The induction of microsomal CYP 2E1 results from enzyme stabilization by alcohol against degradation, and largely proceeds via a posttranscriptional process [283], while the proteasome complex is involved in regulating CYP 2E1 turnover [353]; induction of mitochondrial CYP 2E1 is caused by a different mechanism [353]. Preliminary studies suggest that mitochondrial CYP 2E1 induction may proceed through the stimulation of adenylate cyclase and N-terminal chimeric signals, activated by cyclic AMP-mediated phosphorylation of Ser-129, and supported by protein kinase A [351,353]. Mitochondrial CYP 2E1 further increases after six weeks of alcohol administration, whereas microsomal CYP 2E1 levels off earlier [353]. Some groups favor a more significant role of mitochondrial CYP 2E1 than microsomal CYP 2E1 promoting ALD [351,353,356], whereas other groups are more cautious and prefer microsomal CYP 2E1 as the primary promoting enzyme [234,355,358]. There is also uncertainty as to whether the CYP 2E1-dependent mitochondrial injury is due to ROS, the acetaldehyde generated from ethanol, or both. CYP 2E1 knockout and transgenic models have been developed, which have proven valuable in describing the contributions of overall CYP 2E1 to ALD, and to the toxicity of other toxins [358]. Despite much experimental data on CYP 2E1 being available, clinical studies have provided contradictory results regarding the question to what extent the polymorphism of CYP 2E1 contributes to human ALD, in line with many other risk factors of alcoholic cirrhosis that are under discussion (Figure 21).

A variety of other clinical aspects of CYP 2E1 merit discussion. In brief, and unrelated to alcohol abuse, an increase of CYP 2E1 is observed under various conditions including obesity [138,207,248,269,270,278,281,348] that renders obese individuals more susceptible to liver injury by acetaminophen that is metabolized via CYP 2E1, which appears to play a critical role [25,138,207,283,362]. Other CYP 2E1-dependent conditions relate to diabetes [109,207,352], fasting and acetone [97,107,119,167], nonalcoholic fatty liver [271], and nonalcoholic steatohepatitis [209,270]. In addition, alcohol abuse predisposes the individual to liver injury by carbon tetrachloride, a substrate for the ethanol-induced CYP 2E1; alcoholics are also be more susceptible to renal injury because renal CYP 2E1 may be induced by alcohol [39,257,272,374].

Sufficient evidence exists that microsomal CYP 2E1 likely plays an important role in ALD, more so than mitochondrial CYP 2E1 [252,358]. Based on present knowledge, a real alternative to microsomal CYP 2E1 promoting ALD is not apparent. Chronic alcohol consumption substantially increases microsomal CYP 2E1 in a process called microsomal or ER stress, which is associated with a high production of ROS as important intermediates initiating and perpetuating liver injury, apoptosis, and cell death [245,246,247,248]. ROS attacks various subcellular organelles by initiation lipid peroxidation, leading to a chain reaction that is hardly stopped unless protective compounds such as hepatic glutathione are available in sufficient amounts.

Nuclear genes of Sirtuin-1 (SIRT1) and Sirtuin-2 (SIRT2) are under broad discussion for their potential involvement in various conditions and diseases including ALD [364,365,366] and experimental lifespan extension and the longevity of Japanese centenarians in Okinawa [270]. In short, NAD-dependent deacetylase SIRT1 is involved in the regulation of mitochondrial energy metabolism [364,365,366]. Prolonged alcohol use inhibited hepatic SIRT1 mRNA by 50%, and impaired the protein expression of SIRT1 that was restored by replacing long-chain triglycerides (LCT) with medium chain triglycerides (MCT) in the experimental alcohol diet, suggesting that that mitochondrial dysfunction due to alcohol abuse can be treated by dietary modifications. Other studies support the view that SIRT1 is involved in experimental alcoholic liver injury and overexpressed in human HCC, studied in human HCC cells that retain ADH and CYP 2E1 [365]. Inhibited by alcohol, SIRT1 deregulates hepatic pathways of lipid metabolism such as lipogenesis, fatty acid β-oxidation, and lipoprotein uptake, and pathways of inflammatory response, including expression of pro-inflammatory cytokines [366], substantiating thereby the important role of SIRT1 signaling in the initiation of AFL.

There is growing evidence that in addition to the known direct toxic effects of alcohol and acetaldehyde, immune reactions may partly be responsible for ALD, but the underlying mechanisms are yet poorly understood, and their individual quantitative impact on disease initiation and perpetuation is, to a large part, not well established and a matter of some debate [22,368,369,370]. A partial pathogenetic role of immune and autoimmune reactions in ALD can be assumed because the majority of patients with AH are successfully treated with corticosteroids [25,317,318,319,320,321,322,323,324,325,326,327,328,329,330,331,332,333,334,335], as opposed to a few others who do not benefit from this treatment, possibly due the fact that their liver disease is caused by other mechanisms that are unrelated to an immunity background [25]. Alcohol can modify both, the innate immune system (IIS) and the adaptive immune system (AIS) [370]. In short, IIS is promoted by macrophages, Kupffer cells, neutrophils, and natural killer cells, whereby macrophages are prepared to attack antigens of bacterial cell walls and respond by providing cytokines. Kupffer cells in alcoholic liver disease are specifically activated by lipopolysaccharide (LPS), also called endotoxins, and derived from the outer membrane of Gram-negative intestinal bacteria [370]. 

Endotoxins are considered to play a major pathogenetic role in ALD [348,354,357,371]. New experimental studies revealed that binge alcohol use causes gut leakage and endotoxemia through induction of both intestinal and hepatic CYP 2E1 [357], in support of the microbiome hypothesis that endotoxins produced by intestinal bacteria cross the leaky intestinal mucosa and contribute to hepatic apoptosis and steatohepatitis [354,357], which is still a major clinical issue [348].

Additional discussions focus on how alcohol results in the derangement of metabolic pathways. Among these topics are carotenoids [82,93,103,142,222,367], known for their protective properties against liver injury mediated by ROS [367]. Patients with alcoholic cirrhosis had a 25-fold decreased hepatic level of α- and β-carotene, and more than half of these patients had normal blood levels, suggesting that liver disease modulates intestinal resorption, metabolic pathways, and the removal of α- and β-carotene.

Finally, little attention has been paid in the alcohol literature on the circadian rhythms, which are neglected in virtually all publications that are referenced in this article (Table 3), with the exception of one report published in 1984, describing circadian rhythms of ADH and MEOS in an experimental study [90]. The circadian peak in ADH activity fell near the time of maximal blood ethanol clearance rates both in groups of rats injected with a single ethanol dose (acute group), and in rats continuously exposed to ethanol for 22 weeks (chronic group). However, at all time points, investigated ADH activity levels were lower, and they fluctuated less in the chronic group than in either the acute or control (ethanol naive) groups. In contrast, MEOS activity levels revealed a prominent rhythm that was 180 degrees out of phase with the ADH rhythm in the chronic group, while MEOS activity showed very low levels in the acute and control groups and did not vary over the circadian span. Similarly, alcoholic liver injury is under the control of a molecular circadian clock [373], which is also found in plants and it may have negative effects on their quality [278,279]. Evidence from alcohol studies in experimental animals and clinically in humans presented close associations among circadian processes, alcohol use, and liver injury. In particular, alcohol influences the expression of genes that dominate the circadian clock and regulate metabolic pathways [373]. Consequently, alcohol-based disruption in circadian rhythms may contribute to the initiation and perpetuation of alcoholic liver injury.

## 16. Conclusions

Alcoholic liver disease remains a fascinating topic in clinical and experimental hepatology, and it has gained much input with the discovery of the microsomal ethanol-oxidizing system (MEOS) by Charles S. Lieber and Leonore M. DeCarli, 50 years ago. Some pathogenetic events are linked to acetaldehyde as the first oxidation product that is generated from ethanol, and to cytochrome P450 2E1 as an obligatory component of MEOS. CYP 2E1 produces reactive oxygen species (ROS) such as the ethoxy radical CH_3_CH_2_O, the hydroxyethyl radical CH_3_C(·)HOH, the acetyl radical CH_3_CHO, the singlet radical ^1^O_2_, the superoxide radical HO_2_, hydrogen peroxide (H_2_O_2_), the hydroxyl radical HO, the alkoxyl radical RO, and the peroxyl radical ROO. They attack hepatocytes, Kupffer cells, stellate cells, and liver sinusoidal endothelial cells, and their signaling mediators initiate liver injury, including fibrosis and cirrhosis. Human ALD benefits from appropriate animals, for which experimental models of alcoholic fatty liver are available, allowing for studies on pathogenetic mechanisms. More evidence is now emerging that lipid peroxides, which are generated through ROS and microsomal stress in the membrane of the endoplasmic reticulum, can initiate and perpetuate alcoholic liver injury in joint actions with signaling mediators derived from nonparenchymal cells and hepatocytes. A major pathogenetic role is also assumed for endotoxins that originate from intestinal bacteria and the leaky gut, which results from alcohol-linked modifications of the intestinal microbiome likely as a consequence of hepatic and intestinal CYP 2E1.

Other more recent studies focus on the alcohol-linked inhibition of nuclear genes of Sirtuin-1 (SIRT1). This impairs protein expression and deregulates hepatic pathways, not only of lipid metabolism such as lipogenesis, fatty acid β-oxidation, and lipoprotein uptake, but also of inflammatory responses, including the expression of pro-inflammatory cytokines, thereby substantiating an important role of SIRT1 signaling in the initiation of AFL. The innate immune and adaptive immune systems are modified by alcohol and both could contribute to the individual susceptibility of severe ALD stages, such as AH and AC, which affect only a minority of individuals with alcohol abuse. Finally, and in analogy to the circadian clock in plants, the alcoholic liver is under the control of circadian rhythms that influence many metabolic pathways in the liver, thereby contributing to the natural course of alcoholic liver disease. It is obvious that a unifying mechanism leading to liver injury is not available, but many processes and active mediators are under discussion that may interact among each other. Although these variable pathomechanisms are of interest for biochemists, clinical considerations should focus more on patients with their risk of alcoholic liver injury, and on the early detection of alcoholic fatty liver to prevent disease progression, which is achievable by strict abstinence from alcohol use.

## Figures and Tables

**Table 1:** Differentiation between ADH, MEOS, and catalase. Symbols for effects: (+), marginal; + low; ++, moderate; +++, high; ++++, very high. Abbreviations: ADH, alcohol dehydrogenase; *K*_m_, Michaelis-Menten constant; MEOS, microsomal ethanol-oxidizing system; N.D., not determined. The original table was published in a previous report [25] and is reproduced with permission of the Publisher Taylor & Francis (Didcot, UK).

Characteristics	ADH	MEOS	Catalase
Intracellular localization	Cytosol	Endoplasmic reticulum	Peroxisomes
Co-factor	NAD^+^	NADPH + H^+^	N.D.
Co-substrate	None	Molecular oxygen	H_2_O_2_
Reaction products	Acetaldehyde NADH + H^+^	Acetaldehyde NADP^+^, H_2_O	Acetaldehyde H_2_O
Kinetics			
*K*_m_ (ethanol)	0.5–2.0 mM	7–11 mM	0.6–10 mM
*K*_m_ (O_2_)	N.D.	8.3 μM	50 μM
pH optimum	11	6.9–7.5	5.5
Inhibitory effect			
Pyrazole (0.1 mM)	++++	0	(+)
Cyanide (0.1 mM)	N.D.	0	++++
Azide (0.1 mM)	0	0	++++
Substrate specificity			
Methanol	++	++	++++
Ethanol	+++	++++	++++
n-Propanol	++++	+++	(+)
n-Butanol	++++	++	0
n-Pentanol	++++	+	0
i-Propanol	+	+	0
t-Butanol	0	+	0
Increase in activity following chronic ethanol consumption	0	++++	0
Enzyme isolation	+	+	+
Isoenzymes	+	+	+

**Table 2:** Microsomal ethanol-oxidizing system (MEOS) and its cytochrome P450 isoenzymes. To assess the turnover number, MEOS activity (nmoles acetaldehyde/min) is calculated per nmole cytochrome P450, all expressed per mg of microsomal protein as published [192].

Cytochrome P450 Isoenzyme	MEOS Activity/Cytochrome P450
1A2	10.90
2A6	3.75
2B6	2.89
2D6	0.70
2E1	11.51
3A4	3.38

**Table 3:** Listing of relevant reports addressing issues of MEOS, CYP 2E1, and related aspects.

Year	In Short: Selected Details of MEOS, CYP2E1, and Related Aspects	Authors
1968	Discovery of ethanol oxidation by rat liver microsomal enzymes, now called the hepatic microsomal ethanol-oxidizing system (MEOS), which was different from ADH and catalase, using specific inhibitors, and induction by chronic alcohol consumption	Lieber and DeCarli [26]
1970	Detailed characterization of MEOS	Lieber and DeCarli [27]
1970	Dissociation of MEOS from NADPH oxidase	Lieber and DeCarli [28]
1972	Role of MEOS for ethanol metabolism in vivo	Lieber and DeCarli [29]
1972	Solubilization and purification of MEOS, and its separation from ADH and catalase by DEAE-cellulose ion exchange column chromatography, with the identification of CYP 450, reductase, and phospholipids as components of MEOS	Teschke et al. [30]
1973	Presence of induced MEOS in hepatic smooth and rough microsomes	Ishii et al. [31]
1973	Induced NADPH-cytochrome P450 reductase in hepatic smooth and rough microsomes	Joly et al. [32]
1973	Liver microsomal glycerophosphate acyltransferase activity following prolonged alcohol use	Joly et al. [33]
1973	Increased activity of glucose-6-phosphatase in liver microsomes due to prolonged alcohol consumption	Ishii et al. [34]
1973	A component of hepatic microsomes that is rich in CYP oxidizes ethanol	Mezey et al. [35]
1974	Broad substrate specificity of the microsomal alcohol-oxidizing system (MAOS) for methanol, ethanol, n-propyl alcohol, and n-butyl alcohol, inducible by chronic ethanol consumption	Teschke et al. [36]
1974	MEOS in acatalasemic mice	Lieber and DeCarli [37]
1974	Characterization of the solubilized, isolated, and purified MEOS	Teschke et al. [38]
1974	Enhanced liver injury by carbon tetrachloride after chronic ethanol use: its mechanism	Hasumura et al. [39]
1975	Role of dietary fat and caloric intake for the induction of MEOS by prolonged ethanol use	Joly and Hétu [40]
1975	Alteration of acetaldehyde metabolism after prolonged use of ethanol	Lieber et al. [41]
1975	Detailed description of the microsomal system oxidizing methanol, ethanol, n-propyl alcohol, and n-butyl alcohol as substrates	Teschke et al. [42]
1975	Isolation of the microsomal alcohol-oxidizing system with methanol, ethanol, n-propyl alcohol, n-butyl alcohol, and n-pentanol in normal and acatalasemic mice	Teschke et al. [43]
1975	Inhibition of the ethanol-induced cytochrome P450 by tetrahydrofurane	Ullrich et al. [44]
1975	Ethanol and acetaldehyde metabolism influenced by chronic alcohol use	Lieber et al. [45]
1975	Chronic alcohol consumption decreases acetaldehyde oxidation in liver mitochondria	Hasumura et al. [46]
1976	Characteristics of acetaldehyde oxidation in rat liver mitochondria	Hasumura et al. [47]
1976	Role of MEOS for ethanol metabolism in liver slices, using also n-butyl alcohol as inhibitor	Teschke et al. [48]
1977	Isolation and reconstitution of MEOS, with substrate specificity of the partially purified ethanol-induced CYP 2E1 for ethanol, n-propyl alcohol, and n-butyl alcohol, and characterization of the reconstituted MEOS	Ohnishi and Lieber [49]
1977	Involvement of hydroxyl radicals in MEOS	Cederbaum et al. [50]
1977	Spectral and catalytic properties of an ethanol-induced form of cytochrome P450	Joly et al. [51]
1977	Details of MEOS isolation and reconstitution	Teschke et al. [52]
1977	Current status of MEOS characterization	Teschke et al. [53]
1977	Biochemical nature and role of MEOS	Teschke et al. [54]
1977	MEOS described in Methods in Enzymology	Lieber et al. [55]
1978	Role of superoxide and hydroxyl radicals in MEOS	Ohnishi and Lieber [56]
1978	Reconstitution of MEOS with highly purified microsomal cytochrome P450, reductase, and phospholipids, free of catalase and ADH	Miwa et al. [57]
1978	Photochemical action spectrum of MEOS	Fabry and Lieber [58]
1979	Induction of intestinal MEOS by chronic ethanol administration	Seitz et al. [59]
1979	Induction of MEOS by thyroid hormones	Moreno et al. [60]
1979	Prolonged ethanol use augments liver injury due to paracetamol (acetaminophen)	Teschke et al. [61]
1980	Enhanced chlorpromazine-induced cholestasis following chronic alcohol use	Teschke et al. [62]
1980	Existence and role of MEOS in deermice genetically lacking ADH	Burnett and Felder [63]
1980	Prolonged ethanol use ameliorates liver injury due to dimethylnitrosamine (DMN)	Gellert et al. [64]
1980	Oxidative demethylation of t-butyl alcohol in rat liver microsomes	Cederbaum and Cohen [65]
1980	Thyroid hormones induce MEOS activity and reduce ADH activity in rat liver	Moreno et al. [66]
1981	Microsomal system oxidizing isopropyl alcohol	Cederbaum et al. [67]
1981	Respective role of ethanol and carbohydrates for the induction of MEOS	Teschke et al. [68]
1981	Induction of pulmonary MEOS by chronic ethanol consumption	Seitz et al. [69]
1981	Induction of MEOS by propylthiouracil	Moreno et al. [70]
1981	Prolonged alcohol use potentiates experimental liver injury caused by paracetamol	Sato et al. [71]
1982	Liver enzymes metabolizing ethanol are altered in male rats treated by sex hormones	Teschke and Heymann [72]
1982	Purification and characterization of the ethanol-specific CYP 2E1 in rabbits metabolizing ethanol and aniline	Koop et al. [73]
1982	Substrate specificity of the purified ethanol-induced cytochrome P450 for methanol, ethanol, n-propyl alcohol, n-butyl alcohol, and aniline in rabbits	Morgan et al. [74]
1982	Induction of the ethanol-specific CYP 2E1 by benzene in rabbits	Ingelman-Sundberg and Hagbjörk [75]
1982	Increase of MEOS by a single dose of ethanol	Petersen et al. [76]
1982	Induction of MEOS by testosterone	Teschke and Wiese [77]
1982	Description of the isolated MEOS by electron microscopy and confirmation by method reproduction of the previous description of MEOS regarding its microsomal constituents and independency of ADH and catalase	Damgaard [78]
1982	Decreased hepatic vitamin A levels in patients with ALD	Leo and Lieber [79]
1982	Induction of colonic MEOS by chronic ethanol ingestion	Seitz et al. [80]
1983	Induction of MEOS by hexachlorobenzene	Teschke et al. [81]
1983	Interaction of ethanol with vitamin A	Leo and Lieber [82]
1983	Tumor incidence caused by dimethylnitrosamine is influenced by prolonged alcohol use	Teschke et al. [83]
1983	Liver injury caused by carbon tetrachloride is modified by ethanol administered acutely	Teschke et al. [84]
1983	Liver injury due to chlorpromazine, paracetamol, and dimethylnitrosamine is modified by prolonged use of alcohol	Teschke [85]
1983	MEOS and ethanol metabolism in baboons	Nomura et al. [86]
1983	The alcohol dehydrogenase (ADH) independent pathway of ethanol metabolism in deermice lacking ADH	Shigeta et al. [87]
1984	Induction of the ethanol-specific CYP by imidazole in rabbits	Koop et al. [88]
1984	Induction of the ethanol-specific CYP 2E1 by isoniazid	Gadeholt [89]
1984	Circadian rhythm of MEOS	Sturtevant and Garber [90]
1984	Induction of the ethanol-specific CYP 2E1 by pyrazole in rabbits	Ingelman-Sundberg and Jörnvall [91]
1984	Formation of hydroxyl radical and oxidation of ethanol by CYP 2E1: studies of their mechanisms	Ingelman-Sundberg and Johansson [92]
1984	Reduced liver levels of vitamin A in humans and rats following drug treatment	Leo et al. [93]
1985	Induction of the ethanol-specific CYP 2E1 by trichloroethylene, acetone, pyrazole, and isoniazid in rabbit liver microsomes	Koop et al. [94]
1985	Involvement of the ethanol-specific CYP 2E1 in the microsomal metabolism of dimethylnitrosamine in rats, rabbits, mice, and guinea pigs	Yang et al. [95]
1985	Involvement of the ethanol-specific CYP in the microsomal metabolism of carbon tetrachloride in rabbits	Johansson and Ingelman-Sundberg [96]
1985	Ethanol-inducible CYP 2E1 identified as metabolizing acetone and acetol	Koop and Casazza [97]
1985	Details of liver microsomal CYP induced by isoniazid in the rat	Ryan et al. [98]
1985	Mixed function oxidation in deermice lacking alcohol dehydrogenase: Modification by acute alcohol administration and prolonged consumption of alcohol	Gellert et al. [99]
1986	Studies in deermice containing or missing ADH: Metabolic interactions of ethanol oxidation and mixed-function oxidation	Gellert et al. [100]
1986	Chronic administration of sex hormones and alcohol in female rats and the effect on liver enzymes metabolizing ethanol	Teschke et al. [101]
1986	Microsomal ethanol-oxidizing system of the liver: Biochemical nature and clinical aspects	Teschke [102]
1986	Drugs, retinol, and the relevance of their interactions	Leo et al. [103]
1986	Isoniazid and ethanol: induction of the same microsomal CYP isozyme 3a	Ryan et al. [104]
1986	Ethanol-inducible human liver demethylase for N-nitrosodimethylamine	Wrighton et al. [105]
1986	Ethanol-inducible CYP isozyme in rabbit nasal and kidney microsomes	Ding et al. [106]
1986	Hydroxylation of acetone catalyzed by ethanol- and acetone-inducible CYP in hepatic microsomes and reconstituted membranes	Johansson et al. [107]
1986	Complementary DNA and protein sequences of ethanol-inducible CYPs. A study in rats and humans	Song et al. [108]
1987	Induction of cytochrome P-450j: A study in the spontaneously diabetic BB rat, a strain in which about half of the animals develop insulin-dependent diabetes	Bellward et al. [109]
1987	Purification and characterization of human liver CYP 2E1	Lasker et al. [110]
1987	Hepatic microsomal CYP 2E1 inducible by ethanol in rabbits: Details of cDNA and derived amino acid sequence	Khani et al. [111]
1987	Role of MEOS for interactions with other drugs, carcinogens, and vitamins	Lieber et al. [112]
1987	Pathways contributing to ethanol metabolism: ethanol-metabolizing pathways in deermice. A study on the estimation of flux calculated from isotope effects	Alderman et al. [113]
1988	Ethanol-inducible CYP 2E1 expressed in the centrilobular region of the rat liver	Ingelman-Sundberg et al. [114]
1988	Obesity is considered as a risk factor for drug-induced organ injury: Increased hepatic CYP levels and MEOS activity in the obese overfed rat	Salazar et al. [115]
1988	Acetaldehyde adducts formed with ethanol-inducible CYP 2E1 in vivo	Behrens et al. [116]
1988	CYP 2E1 in rabbit olfactory mucosa: its induction by ethanol and acetone	Ding et al. [117]
1988	Ethanol-inducible CYP 2E1: a study on molecular regulation in hamsters	Kubota et al. [118]
1988	Metabolism of benzene in microsomes obtained from rat and rabbit liver, and the role of CYP 2E1 induced by ethanol, acetone, and benzene	Johansson and Ingelman-Sundberg [119]
1988	Ligand-dependent maintenance of ethanol-inducible CYP: an experimental study using primary rat hepatocyte cell cultures	Eliasson et al. [120]
1988	Hepatic microsomal ethanol-inducible CYP 2E1 and its intralobular distribution of in liver	Tsutsumi et al. [121]
1988	Prolonged alcohol use enhances oxygen radical dependent inactivation of metabolic enzymes by liver microsomes	Dicker and Cederbaum [122]
1989	Induction and tissue-specific expression of rabbit *CYP 2E1* genes	Porter et al. [123]
1989	The intralobular distribution of ethanol-inducible CYP 2E1; an experimental study in rat liver and a clinical analysis in human liver	Tsutsumi et al. [124]
1990	Ethanol-inducible CYP 2E1 and its regional distribution of in the central nervous system: an experimental study in rats	Hansson et al. [125]
1990	Modification of hepatic CYP 2E1 by pituitary hormones in rats and mice	Hong et al. [126]
1990	Ethanol-inducible CYP 2E1: multiple mechanisms are involved in its regulation	Koop and Tierney [127]
1990	Lymphocytes from patients with poorly controlled insulin-dependent diabetes exhibit increased CYP 2E1	Song et al. [128]
1990	Induction of rat hepatic CYP 2E1 by pyridine	Kim et al. [129]
1990	Solvents enhance the translational efficiency in the course of CYP 2E1 induction	Kim et al. [130]
1990	CYP 2E1 influences the interactions of ethanol with enflurane metabolism and their toxicity	Tsutsumi et al. [131]
1990	CYP 2E1 is involved in nitrosamine metabolism and regulation	Yang et al. [132]
1990	Chlorzoxazone hydroxylation is a specific probe for CYP 2E1 in human liver	Peter et al. [133]
1990	Localization of ethanol-inducible CYP 2E1 assessed by immunohistochemistry in the alimentary tract of rats	Shimizu et al. [134]
1991	Post-translational reduction of CYP 2E1 by CCl_4_	Sohn et al. [135]
1991	Role of hormones in the phosphorylation and degradation of CYP 2B1 and 2E1: a study in in isolated rat hepatocytes	Johansson et al. [136]
1991	Dietary lipids and carbohydrates modify the levels of CYP 2E1 in microsomes obtained from rat liver	Yoo et al. [137]
1991	CYP 2E1 is induced in the in the experimental obese rat model	Raucy et al. [138]
1991	Acetaldehyde as another substrate for ethanol-inducible CYP 2E1	Terelius et al. [139]
1991	Identification and induction of CYP 2E1 in Kupffer cells of an experimental model of rats	Koop et al. [140]
1991	Genetic polymorphism in the 5′-flanking region change transcriptional regulation of the *CYP 2E1* gene: a study in humans	Hayashi et al. [141]
1992	Interaction of ethanol with β-carotene: Delayed blood clearance and evidence of increased liver injury	Leo et al. [142]
1992	Intracellular degradation of CYP 2E1 is controlled by hormones and substrates	Eliasson et al. [143]
1992	Distribution the ethanol-inducible CYP 2E1 in the pancreas of rats fed ethanol combined with high fat or low fat diet	Sohda et al. [144]
1992	Oxidative and reductive metabolic pathways by CYP 2E1	Koop [145]
1992	CYP 2E1 and 2A6 enzymes are the preferred catalysts for metabolic activation of N-nitrosodialkylamines and nitrosamines in the microsomes of human liver	Yamazaki et al. [146]
1993	Inhibition of chlorzoxazone metabolism by a single dose of disulfiram, and its potential role as a clinical probe for CYP 2E1	Kharasch et al. [147]
1993	Enflurane defluorination catalyzed by CYP 2E1 in microsomes of human liver	Thummel et al. [148]
1993	Human CYP 2E1 stability in HepG2 cells	Day et al. [149]
1993	*Dra*I and *Rsa*I restriction fragment length polymorphisms analyzed in a study from Finland	Hirvonen et al. [150]
1993	Pathogenesis of alcoholic liver disease and the role of CYP 2E1	Morimoto et al. [151]
1993	NADPH- and NADH-dependent production of superoxide and hydroxyl radical is enhanced in hepatic microsomes obtained following prolonged alcohol use	Rashba-Step et al. [152]
1993	Increased enzyme synthesis is responsible for the in vivo induction of hepatic CYP 2E1	Tsutsumi et al. [153]
1993	Formation of 19(S)-, 19(R)-, and 18(R)-hydroxyeicosatetraenoic acids by alcohol-inducible CYP 2E1	Laethem et al. [154]
1993	Induction of CYP 2E1 during prolonged alcohol use is due to the transcription of the *CYP 2E1* gene when blood alcohol concentrations are high	Badger et al. [155]
1993	Contribution of cytochrome P-450s to MEOS: assessed by a specific and sensitive assay of MEOS activity using HPLC with fluorescence labeling	Kunitoh et al. [156]
1993	Levels of CYP 1A2 and CYP 2E1, and their related monooxygenase activities in human liver obtained as surgical samples	Lucas et al. [157]
1993	CYP 2E1 induction during chronic ethanol exposure occurs by a two-step mechanism, depending on blood alcohol levels: a study in rats	Ronis et al. [158]
1993	CYP 2E1 is the preferred enzyme catalyzing the defluorination of sevoflurane, isoflurane, and methoxyflurane in human liver microsomes	Kharasch et al. [159]
1993	Inhibition of CYP 2E1 by ethanol is caused in the human liver by corresponding increase in encoding messenger RNA	Takahashi et al. [160]
1993	Use of 4-nitrophenol as an in vitro substrate probe was validated for human liver CYP 2E1	Tassaneeyakul et al. [161]
1994	Alcohol-derived radicals and their spin trapping in liver microsomes and reconstituted systems	Albano et al. [162]
1994	Ethanol augments the content and activity of human CYP 2E1 in a transduced HEPG2 cell line	Carrocio A [163]
1994	Significance of tissue-specific expression and methylation of the human *CYP 2E1* gene	Botto et al. [164]
1994	Role of genetic CYP 2E1 polymorphism for the development of alcoholic liver disease	Tsutsumi et al. [165]
1994	Ethnic variation in the *CYP 2E1* gene: Polymorphism analysis of 695 African-Americans, European-Americans and Taiwanese	Stephens et al. [166]
1994	Relationship between CYP 2E1 and acetone catabolism in rats as studied with the inhibitor diallyl sulfide	Chen et al. [167]
1994	Association between restriction fragment-length polymorphism of the human *CYP 2E1* gene and susceptibility to alcoholic liver cirrhosis	Maezawa et al. [168]
1994	Involvement of CYP 2E1 in the (omega-1)-hydroxylation of lauric acid in rat liver microsomes	Amet et al. [169]
1994	CYP 2E1 induction by ethanol in a rat hepatoma FGC-4 cell model	McGehee et al. [170]
1994	Piperine modifies the expression of P4502E1, P4502B, and P4501A in rats	Kang et al. [171]
1994	Restriction fragment-length polymorphism of the human *CYP 2E1* gene and susceptibility to lung cancer: Possible relevance to low smoking exposure	Uematsu et al. [172]
1994	Differences of regulation and expression of the human *CYP 2E1* gene due to the *Rsa*I polymorphism in the 5’ flanking region	Watanabe et al. [173]
1995	An *Rsa*I polymorphism in the *CYP 2E1* gene does not affect lung cancer risk in a Japanese population	Watanabe et al. [174]
1995	Ethanol induces CYP 2E1 by a mechanism involving protein stabilization	Roberts et al. [175]
1995	Renal tumorigenicity of 1,1-dichloroethene in mice: the role of male-specific expression of CYP 2E1 in the renal bioactivation of 1,1-dichloroethene	Speerschneider and Dekant [176]
1995	Intestinal toxicity of acrylonitrile: in vitro metabolism by intestinal CYP 2E1	Subramanian and Ahmed [177]
1995	Stable expression of human CYP 2E1 in V79 Chinese hamster cells	Schmalix et al. [178]
1995	Lacking association of polymorphism at the CYP 2E1 locus with alcoholic liver disease in Caucasian men	Carr et al. [179]
1995	Modulation of experimental alcoholic liver injury by inhibitors of CYP 2E1	Morimoto et al. [180]
1995	Genetic polymorphism of CYP 1A1, 2D6 and 2E1: Regulation and toxicological significance	Rannug et al. [181]
1995	Genetic polymorphism of CYP 2E1 and risk of alcoholic liver disease in Caucasians	Pirmohamed et al. [182]
1995	*CYP 2E1* genotype and chlorzoxazone metabolism in healthy and alcoholic Caucasians	Lucas et al. [183]
1995	Decreased CYP 2E1 as assessed by the rate of chlorzoxazone hydroxylation in alcoholics during the withdrawal phase	Lucas et al. [184]
1995	Respective roles of CYP 2E1 and CYP A2 in chlorzoxazone, and ethanol metabolism in mammalian liver microsomes	Mishin et al. [185]
1995	CYP 2E1 is not the sole catalyst of chlorzoxazone hydroxylation in rat liver microsomes	Jayyosi et al. [186]
1995	Selectivity of CYP 2E1 in catalyzing chlorzoxazone 6-hydroxylation	Yamazaki et al. [187]
1995	Insulin down-regulates CYP 2B and 2E expression at the posttranscriptional level in the rat hepatoma cell line	De Waziers et al. [188]
1995	Evidence for a tissue-specific induction of cutaneous CYP 2E1 by dexamethasone	Sampo et al. [189]
1995	Ethanol oxidizing enzymes: Roles in alcohol metabolism and alcoholic liver disease	Crabb [190]
1995	CYP 2E1 changes in rat liver, kidney and lung microsomes after prolonged alcohol application, either orally or by inhalation	Zerilli et al. [191]
1996	Microsomal ethanol oxidizing system activity by human hepatic cytochrome P-450s and involvement of CYP 1A2, 2A6, 2B6, 2D6, 2E1, and 3A4	Asai et al. [192]
1996	High inducibility of mouse renal *CYP 2E1* gene by tobacco smoke and its possible effect on DNA single strand breaks	Seree et al. [193]
1996	Effects of diet and ethanol on the expression and localization of CYP 2E1 and 2C7 in the colon of male rats	Hakkak et al. [194]
1996	Induction of CYP 2E1 by ethanol in rat Kupffer cells	Koivisto et al. [195]
1996	Human CYP 2E1: From genotype to phenotype	Carriere et al. [196]
1996	Expression, catalytic activity, and inducibility of CYP 2E1 in the rat central nervous system	Tindberg and Ingelman-Sundberg [197]
1997	Enzymatic degradation of chlorzoxazone by hepatic microsomes from humans and 10 other mammalian species	Court et al. [198]
1997	Regulation of the hepatic *CYP 2E1* gene during prolonged ethanol exposure: Lack of an ethanol response element in the proximal 5′-flanking sequence	McGehee et al. [199]
1997	Immunohistochemical determination of hepatic CYP 2E1 in formalin-fixed, paraffin-embedded sections	Cohen et al. [200]
1997	Effect of fatty acids and ketone bodies on CYP 2B, 4A, and 2E1 expression in primary cultured rat hepatocytes	Zangar and Novak [201]
1997	Ethanol metabolism in the brain	Zimatkin and Deitrich [202]
1997	Inhibition of CYP 2E1 expression by 2-(allylthio) pyrazine, a potential chemoprotective agent, and considerations on hepatoprotective effects	Kim et al. [203]
1997	Insulin effects on CYP 2E1, 2B, 3A, and 4A expression in primary cultured rat hepatocytes	Woodcroft and Novak [204]
1997	Lipid peroxidation, CYP 2E1 and arachidonoid acid metabolism in alcoholic liver disease in rats	French et al. [205]
1997	Chlormethiazole inhibition of CYP 2E1 as assessed by chlorzoxazone hydroxylation in humans	Gebhardt et al. [206]
1998	CYP 2E1 activity as assessed by chlorzoxazone hydroxylation: studies in patients with diabetes and obesity	Lucas et al. [207]
1998	Expression of CYP 2E1 in human liver: Assessment by mRNA, genotype, and phenotype	Powell et al. [208]
1998	Increased hepatic CYP 2E1 in patients with nonalcoholic steatohepatitis	Weltman et al. [209]
1998	Respective roles of human CYP 2E1 and 3A4 in the hepatic microsomal ethanol oxidizing system	Salmela et al. [210]
1998	Microsomal acetaldehyde oxidation is negligible in the presence of ethanol	Wu et al. [211]
1998	Selective inhibition of CYP 2E1 in vivo and in vitro with trans-1, 2-dichloroethylene	Matthews et al. [212]
1998	CYP 2E1 and its catalytic activity in rat testis	Jiang et al. [213]
1998	CYP 2E1 and 1A1 in the rat pancreas	Kessova et al. [214]
1998	CYP 2E1 is present in the rat pancreas and induced by prolonged alcohol consumption	Norton et al. [215]
1998	Polyenylphosphatidylcholine opposes the increase of CYP 2E1 by ethanol, and corrects the iron-induced decrease	Aleynik et al. [216]
1998	Involvement of CYP 2E1 in the (omega-1)-hydroxylation of oleic acid in human and rat liver microsomes	Adas et al. [217]
1998	Chlorzoxazone pharmacogenetics, a potential marker of hepatic CYP 2E1 in humans	Mishin et al. [218]
1998	Inhibition of CYP 2E1 by chlormethiazole as measured by chlorzoxazone pharmacokinetics in patients with alcoholism and in healthy volunteers	Eap et al. [219]
1998	Regulation of rabbit CYP 2E1 expression in HepG2 cells by insulin and thyroid hormones	Peng and Coon [220]
1998	CYP 2E1 inducibility and hydroxyethyl radical formation among alcoholics	Dupont et al. [221]
1999	Alcohol, vitamin A, and beta-carotene: adverse interactions, including hepatotoxicity and carcinogenicity	Leo and Lieber [222]
1999	Expression of CYP 2E1 by human monocyte-derived macrophages	Hutson and Wickramasinghe [223]
1999	Carbon monoxide, cigarette smoking, and CYP 2E1 activity	Benowitz et al. [224]
1999	Chlorzoxazone, a selective probe for phenotyping CYP 2E1 in humans	Lucas et al. [225]
2001	Effects of alcohol and diallylsulphide on CYP 2E1 activity in humans: a phenotyping study using chlorzoxazone	Loizou and Cocker [226]
2001	Inhibition of CYP 2E1 with natural agents may be a feasible strategy for minimizing liver injury by ethanol	McCarty [227]
2001	Ethanol and oxidative stress	Sun et al. [228]
2002	Effect of chronic disulfiram administration on CYP 1A2, CYP 2C19, CYP 2D6, CYP 2E1, and N-acetyltransferase in healthy humans	Frye and Branch [229]
2003	Rapid determination of enzyme activities of recombinant human CYPs, human liver microsomes, and hepatocytes	Ghosal et al. [230]
2004	CYP 2E1: biochemistry, toxicology, regulation, and function in alcoholic liver injury	Kessova and Cederbaum [231]
2004	Robustness of chlorzoxazone as an in vivo measure of CYP 2E1 activity	Ernstgard et al. [232]
2006	Effect of high-dosed aspirin on CYP 2E1 in healthy humans measured using chlorzoxazone as a probe	Park et al. [233]
2008	CYP 2E1 and oxidative liver injury caused by alcohol	Choi et al. [234]
2010	CYP-mediated differential oxidative modification of proteins: Albumin, apolipoprotein E, and CYP 2E1 as targets	Wellman and Siest [235]
2014	Association studies of CYP, family 2, subfamily E, and polypeptide 1 (CYP 2E1) gene polymorphisms with acute rejection in kidney transplantation recipients	Kim et al. [236]
2014	Pathogenesis of alcoholic liver disease: Significance of oxidative metabolism	Ceni et al. [237]
2016	Resveratrol pretreatment affects CYP 2E1 activity in healthy volunteers	Bedada and Neerati [238]
2017	Effect of piperine on CYP 2E1 enzyme activity in healthy volunteers	Bedada and Boga [239]
2017	The role of human CYP 2E1 in liver inflammation and fibrosis	Xu et al. [240]
2017	CYP 2E1 is involved in aging-related kidney damage in mice through increased nitroxidative stress	Abdelmegeed et al. [241]
2018	Vinyl chloride, CYP 2E1, and liver injury	Fujiwara [242]
2018	Vinyl chloride, diet, and liver injury	Lang et al. [243]

Abbreviations: ADH, Alcohol dehydrogenase; MEOS, Microsomal ethanol-oxidizing system.

**Table 4:** Alcoholic liver disease and the 5-hit working hypothesis with a tentative cascade of events. Hypothetical steps of the five hits leading to end-stage alcoholic liver disease. Adapted from a previous report [25] and reproduced with permission of the PublisherTaylor & Francis (Didcot, UK).

**First Hit.**	The first hit is dependent on ADH and occurs at low alcohol levels through the generation not only of NADH + H^+^ leading to an increased NADH + H^+^/NAD^+^ ratio, which stimulates hepatic fatty acid synthesis [22] and increases α-glycerophosphate-trapping fatty acids [22,33], but also of acetaldehyde, which impairs hepatic mitochondrial functions including hepatic mitochondrial fatty acid oxidation [22]. This first hit fully explains at least in part the development of alcoholic fatty liver.
**Second Hit.**	The second hit is classified as a transition from alcoholic fatty liver to alcoholic steatohepatitis, most likely triggered by the increased production of acetaldehyde via MEOS [22,23] and of reactive oxygen species (ROS) with its capacity for irreversible covalently binding to cellular macromolecules, including membrane proteins and phospholipids [45,50,56,92,152,205,231,234,237,244,245,246,247,248,249,250,251,252]. These injurious alterations at the molecular and cellular level cause some necrosis, apoptosis, and inflammatory cells in the fatty liver, justifying the term alcoholic steatohepatitis, as it includes toxic hepatitis in steatosis [25]. Further stages are characterized by perisinusoidal and pericentral fibrosis due to participation of non-hepatocytes such as Kupffer cells, stellate cells, and sinus endothelial cells. Mediators such growth factors, interferons, interleukins, tumor necrosis factor and endotoxins, as well as hepatic iron, are considered as possible active promoters of liver injury, but considering the multiplicity of proposed mediators, it is difficult to predict how they interact with each other and modify the course of liver injury.
**Third Hit.**	The third hit initiates a more severe liver injury stage, whereby alcoholic steatohepatitis is the precursor in most, but certainly not all patients with alcoholic hepatitis. Steatosis is no more a characteristic feature, but is now replaced by necrosis, apoptosis, and inflammation. At this stage, injury becomes more severe and presents with more fibrosis and as a self-perpetuating process, immunity aspects gain additional relevance, because alcohol modifies the innate and adapted immune system, which may explain the individual differences of susceptibility for ALD. With the third hit, the disease may approach a point of no return.
**Fourth Hit.**	The fourth hit is dominated by increased fibrosis, due to increased collagen formation. This allows for a clinically unrecognizable transition from alcoholic hepatitis with fibrosis to irreversible cirrhosis. However, AC can also develop without ASH or AH.
**Fifth Hit.**	In rare cases, a fifth hit initiates the development of a hepatocellular carcinoma (HCC), mostly occurring in patients with cirrhosis. This final hit scenario of carcinogenesis is triggered by acetaldehyde and ROS through the generation of DNA adducts, which promote mutagenesis, and interference with methylation, synthesis, and repair of DNA. Suggested is a possible role of SIRT1. These overall events will enhance AHCC susceptibility, keeping in mind that ethanol itself is not a carcinogenetic chemical.

**Table 5:** Potentially toxic metabolites resulting from the enzymatic degradation of ethanol in the liver Derived from original reports and review articles [25,50,56,92,152,205,231,237,245,246,247,248,249,250,251,252].

Selected Potentially Toxic Metabolites and Reactive O_2_-Species due to Hepatic Ethanol Degradation
Acetaldehyde C_2_H_4_O
Ethoxy radical CH_3_CH_2_O
Hydroxyethyl radical CH_3_C(·)HOH
Acetyl radical CH_3_CHO
Singlet radical ^1^O_2_
Superoxide radical HO_2_
Hydrogen peroxide H_2_O_2_
Hydroxyl radical HO
Alkoxyl radical RO
Peroxyl radical ROO
Lipid peroxides

**Table 6:** GGT activities in AFL. Studied were 19 patients with AFL and nine patients without AFL. Animals: eight rats with AFL, fed chronically with an alcohol-containing diet were compared with eight controls that were fed control diets. Data are given as means ± SD, adapted from a previous report [295]. Abbreviations: AFL, Alcoholic fatty liver; GGT, gamma-glutamyltransferase.

Study Cohort	Alcoholic Fatty Liver	Controls	Significance
**Patients**			
Serum GGT (U/L)	195.0 ± 93.7	13.7 ± 2.0	*p* < 0.025
Liver GGT			
(U/g wet weight)	4.78 ± 0.4	1.91 ± 0.2	*p* < 0.025
(U/g protein)	35.9 ± 16.1	16.4 ± 6.6	*p* < 0.025
**Animals**			
Serum GGT (U/L)	4.41 ± 1.64	2.19 ± 0.31	*p* < 0.025
Liver GGT			
(U/g wet weight)	0.14 ± 0.06	0.07 ± 0.03	*p* < 0.001
(U/g protein)	1.19 ± 0.23	0.79 ± 0.19	*p* < 0.0.25
(U/100 g body weight)	0.80 ± 0.28	0.34 ± 0.09	*p* < 0.001

**Table 7:** Serum AST, ALT, and GDH activities in alcoholic fatty liver.

Patient with AFL	Degree of Steatosis	AST (U/L)	ALT (U/L)	Ratio AST/ALT	GDH (U/L)
1	50%	12.4	17.7	0.70	53.9
2	60%	100.7	22.5	4.47	35.8
3	60%	50.3	19.9	2.52	16.8
4	80%	54.7	9.2	5.94	7.7
5	10–15%	20.6	31.7	0.65	2.0
6	50%	25.6	33.9	0.76	7.9
7	60–70%	61.4	62.4	0.98	9.1
8	60–70%	11.6	7.9	1.47	1.8
9	30–40%	33.2	61.1	0.54	4.1
10	80%	53.6	19.0	2.82	1.9
11	80–90%	34.6	46.8	0.74	9.2
12	30%	16.0	30.6	0.52	3.3
13	30–40%	32.9	19.9	1.65	4.8
14	20–30%	11.3	33.8	0.33	7.3
15	50–60%	25.7	62.2	0.41	7.2
16	10%	70.1	19.3	3.63	7.2
17	10–15%	9.2	6.7	1.37	0.8
18	50%	26.1	68.1	0.38	7.5
19	50%	10.9	9.2	1.19	2.1
Means ± SEM		34.8 ± 5.7	30.2 ± 4.6	1.53 ± 1.51	9.9 ± 3.0

Normal range was for AST < 35 U/L, ALT < 45 U/L, and GDH < 7 U/L. Abbreviations: AST, Aspartate aminotransferase; ALT, Alanine aminotransferase; GDH, Glutamate dehydrogenase. Results are derived from 19 patients with alcoholic fatty liver, and with some case details published previously [295].

## Abbreviations

ABIC	Age, bilirubin, INR, and creatinine score
AASLD	American Association of the Study of Liver Diseases
AC	Alcoholic cirrhosis
ADH	Alcohol dehydrogenase
AFL	Alcoholic fatty liver
ALD	Alcoholic liver disease
ALDH	Acetaldehyde dehydrogenase
AH	Alcoholic hepatitis
AHCC	Alcoholic hepatocellular carcinoma
ALT	Alanine transaminase
ASH	Alcoholic steatohepatitis
AST	Aspartate transaminase
CDT	Carbohydrate-deficient transferrin
CLD	Chronic liver diseases
CYP	Cytochrome P450
CYP2E1	Cytochrome P450 2E1
DILI	Drug induced liver injury
EASL	European Association for the Study of the Liver
FPM	First pass metabolism
GAHS	Glasgow Alcoholic Hepatitis Score
GDH	Glutamate dehydrogenase
GGT	Gamma-glutamyltransferase
HBV	Hepatitis B virus
HCV	Hepatitis C virus
HILI	Herb-induced liver injury
*K*_m_	Michaelis-Menten constant
MCV	Mean corpuscular volume of erythrocytes
MDB	Mallory-Denk bodies
MDF	Maddrey Discriminant Function scale
MELD	Model for End-stage Liver Disease score
MEOS	Microsomal ethanol-oxidizing system
MAST	Michigan Alcoholism Screening Test
NAFLD	Nonalcoholic fatty liver disease
NASH	Nonalcoholic steatohepatitis
PTU	Propylthiouracil
ROS	Reactive oxygen species
RUCAM	Roussel Uclaf Causality Assessment Method
